# Impact of early events and lifestyle on the gut microbiota and metabolic phenotypes in young school-age children

**DOI:** 10.1186/s40168-018-0608-z

**Published:** 2019-01-04

**Authors:** Huanzi Zhong, John Penders, Zhun Shi, Huahui Ren, Kaiye Cai, Chao Fang, Qiuxia Ding, Carel Thijs, Ellen E. Blaak, Coen D. A. Stehouwer, Xun Xu, Huanming Yang, Jian Wang, Jun Wang, Daisy M. A. E. Jonkers, Ad A. M. Masclee, Susanne Brix, Junhua Li, Ilja C. W. Arts, Karsten Kristiansen

**Affiliations:** 1 BGI-Shenzhen, Shenzhen, 518083 China; 2 China National GeneBank, BGI-Shenzhen, Shenzhen, 518120 China; 3 Laboratory of Genomics and Molecular Biomedicine, Department of Biology, University of Copenhagen, 2100 Copenhagen, Denmark; 4 Department of Medical Microbiology, NUTRIM School of Nutrition and Translational Research in Metabolism & Care and Public Health Research Institute CAPHRI, Maastricht University Medical Centre, Maastricht, the Netherlands; 5 Department of Epidemiology, Care and Public Health Research Institute CAPHRI, Maastricht University, Maastricht, the Netherlands; 6 Department of Human Biology, NUTRIM School of Nutrition and Translational Research in Metabolism, Maastricht University Medical Centre, Maastricht, the Netherlands; 7 Department of Internal Medicine, CARIM School for Cardiovascular Diseases, Maastricht University Medical Centre, Maastricht, The Netherlands; 8 James D. Watson Institute of Genome Sciences, Hangzhou, 310058 China; 9 Division of Gastroenterology-Hepatology, Department of Internal Medicine, NUTRIM School of Nutrition, Toxicology and Metabolism, Maastricht University Medical Centre, Maastricht, the Netherlands; 10 Department of Biotechnology and Biomedicine, Technical University of Denmark, Soltofts Plads, 2800 Kongens Lyngby, Denmark; 11 School of Bioscience and Biotechnology, South China University of Technology, Guangzhou, 510006 China; 12 Maastricht Centre for Systems Biology (MaCSBio) & Department of Epidemiology, CARIM School for Cardiovascular Diseases, Maastricht University, Maastricht, the Netherlands

**Keywords:** School-age children, Gut microbiota, Enterotype, Metabolic phenotypes

## Abstract

**Background:**

The gut microbiota evolves from birth and is in early life influenced by events such as birth mode, type of infant feeding, and maternal and infant antibiotics use. However, we still have a gap in our understanding of gut microbiota development in older children, and to what extent early events and pre-school lifestyle modulate the composition of the gut microbiota, and how this impinges on whole body metabolic regulation in school-age children.

**Results:**

Taking advantage of the KOALA Birth Cohort Study, a long-term prospective birth cohort in the Netherlands with extensive collection of high-quality host metadata, we applied shotgun metagenomics sequencing and systematically investigated the gut microbiota of children at 6–9 years of age. We demonstrated an overall adult-like gut microbiota in the 281 Dutch school-age children and identified 3 enterotypes dominated by the genera *Bacteroides*, *Prevotella*, and *Bifidobacterium*, respectively. Importantly, we found that breastfeeding duration in early life and pre-school dietary lifestyle correlated with the composition and functional competences of the gut microbiota in the children at school age. The correlations between pre-school dietary lifestyle and metabolic phenotypes exhibited a striking enterotype dependency. Thus, an inverse correlation between high dietary fiber consumption and low plasma insulin levels was only observed in individuals with the *Bacteroides* and *Prevotella* enterotypes, but not in *Bifidobacterium* enterotype individuals in whom the gut microbiota displayed overall lower microbial gene richness, alpha-diversity, functional potential for complex carbohydrate fermentation, and butyrate and succinate production. High total fat consumption and elevated plasma free fatty acid levels in the *Bifidobacterium* enterotype are associated with the co-occurrence of *Streptococcus*.

**Conclusions:**

Our work highlights the persistent effects of breastfeeding duration and pre-school dietary lifestyle in affecting the gut microbiota in school-age children and reveals distinct compositional and functional potential in children according to enterotypes. The findings underscore enterotype-specific links between the host metabolic phenotypes and dietary patterns, emphasizing the importance of microbiome-based stratification when investigating metabolic responses to diets. Future diet intervention studies are clearly warranted to examine gut microbe-diet-host relationships to promote knowledge-based recommendations in relation to improving metabolic health in children.

**Electronic supplementary material:**

The online version of this article (10.1186/s40168-018-0608-z) contains supplementary material, which is available to authorized users.

## Introduction

Microbial colonization in early life is crucial for infant health and may affect health status in later life [1, 2]. Substantial effort has been devoted into studying the development of the gut microbiota during infancy. Throughout the first year of life, the gut microbiota increases dramatically in diversity and stability, and reportedly reaches an adult-like configuration in the subsequent years [3–5]. Many studies have demonstrated that early events such as birth mode, type of infant feeding, the presence of older siblings, and maternal and infant antibiotics use affect the establishment and composition of gut microbiota during infancy [4, 6–8]. After weaning, dietary patterns moreover have a profound impact on shaping the childhood gut microbiota [9]. However, we still have a gap in knowledge of the roles played by early events and lifestyle in the development of the gut microbiota during childhood.

Studies conducted with animal models and adult human beings have provided strong indications of metabolic cross-talk between gut microbes and the host. The gut microbiota influences the development and regulation of the immune system, and energy and metabolic homeostasis of the host via the production of a vast array of metabolites such as short-chain fatty acids (SCFAs) and secondary bile acids [10–12]. For instance, SCFAs produced by gut bacterial fermentation of complex dietary carbohydrates interact with G protein-coupled receptors (GPCRs) and affect adiposity and insulin resistance [13]. Nevertheless, our understanding of how dietary patterns interact with the gut microbiota and subsequently affect metabolic traits of children is limited.

In this study, we examined the composition and functional potential of the gut microbiota of 281 Dutch children at early school-age (6–9 years of age) and revealed the impact of early events and pre-school dietary patterns on the gut microbiota of children. We identified enterotype-based differences of not only the structure and functional potential of the microbial communities, but also differences in the correlations between metabolic phenotypes and dietary patterns in children. Our results provide new insights into the normal developmental trajectories of the gut microbiota and the environmental factors affecting microbiota development in school-age children, and increase our understanding of the microbiota-dependent interactions between diets and host metabolic phenotypes.

## Results

### Characterizing the gut microbiome of early school-age Dutch children

**Fig. 1:**
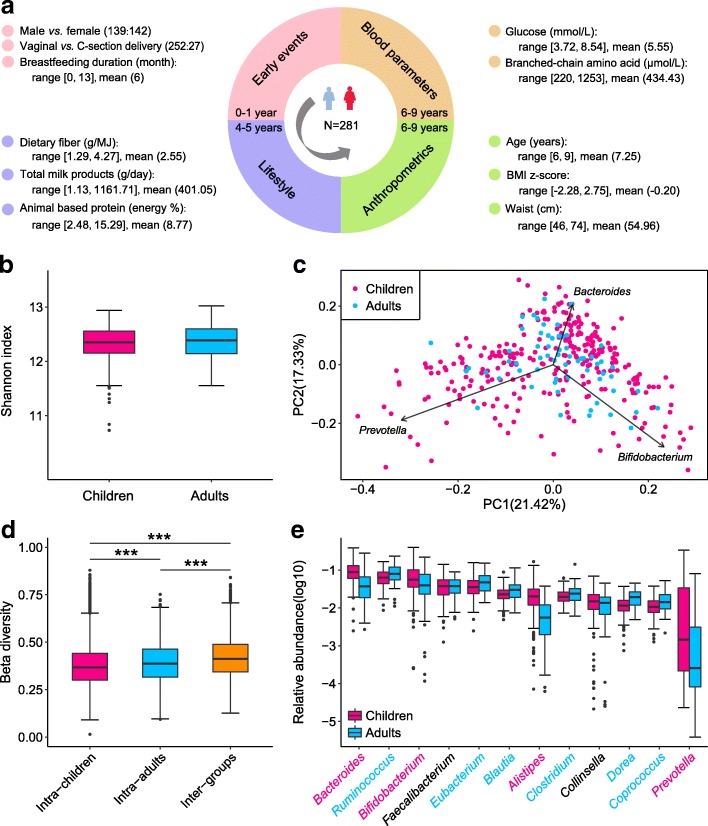
Comparison between early school-age Dutch children and adults. **a** Categories of phenotypic data collected within the KOALA cohort. **b** Box plot showing the gene-based α-diversity (Shannon index) in early school-age Dutch children (*n* = 281) and healthy Dutch adults (*n* = 62). **c** Genus-based principal component analysis (PCA) of children and adults. **d** Box plots of intra- and inter-group beta diversity based on genus profiles in children and adults. The “Intra-children” and “Intra-adults” indicate the genus-based beta diversity in children and adults, and the “Inter-groups” indicates the genus-based beta diversity between children and adults (***P < 0.001; Wilcoxon rank-sum test). **e** Box plots displaying the ten most abundant genera among children and adults. Genera indicated with red font are enriched in children, and genera in blue are enriched in adults. Boxes are ordered according to median relative abundance of genus in children

To investigate the gut microbial characteristics of healthy early school-age Dutch children as well as the relationship between their gut microbiota and multiple phenotypic parameters, we collected stool samples from 281 children at 6–9 years of age (mean age 7.3 years) enrolled in the KOALA Birth Cohort Study [14], subjected the samples to shotgun metagenomic sequencing, and analyzed the results against additional measures of 45 phenotypic parameters classified into four categories: (1) early events; (2) pre-school lifestyle, including diet, both collected through repeated questionnaires up to 5 years of age; (3) blood parameters collected in parallel with fecal samples at 6–9 years of age; and (4) anthropometric measurements collected at both 4–5 years and 6–9 years of age (Fig. 1a, Additional file 1: Table S1). After performing shotgun metagenomic sequencing and quality control, we acquired a total of 1.28 Tb of high-quality non-human clean reads, corresponding to 49.24 million reads per child. Genes were identified by aligning the clean reads to the 9.9M human gut microbiome integrated gene catalog (IGC) [15]with an average of 80.1% reads in each sample being mapped (Additional file 1: Table S2). Screening the Maastricht Irritable Bowel Syndrome Cohort (MIBS-CO) [16] for healthy adult controls who fitted our inclusion criteria provided 62 metagenomic datasets from healthy Dutch adults for our age-based comparison (Additional file 1: Table S3). We applied the same pipeline as used for processing the children’s samples to analyze the published adult samples, resulting in 26.9 million clean reads per adult and a 77.4% IGC mapping ratio per adult.

### Comparison of gut microbiota in Dutch children and adults, and overweight and lean children

Comparison of gut microbial gene diversity in Dutch adults and children revealed an adult-like alpha diversity in the school-age children (Wilcoxon rank-sum test, *P* > 0.05, Fig. 1b). Principal component analysis (PCA) based on genus profiles showed no separation between Dutch children and adults (Fig. 1c). Within- and between-group β diversity of genus profiles further revealed a slightly more similar microbial composition within children compared to within adults (Fig. 1d). In line with previous studies comparing healthy infants, children (1–12 years), and adults [3, 17, 18], Dutch children showed a relative enrichment in abundance of the genus *Bifidobacterium* compared to adults (adjusted *P* < 0.05, Fig. 1e, Additional file 1: Table S4). Of note, the abundance of *Bifidobacterium adolescentis*, a reported adult-type *Bifidobacterium* species with no or poor ability for human milk oligosaccharides (HMOs) degradation [9, 19, 20], showed no differences between children and adults (Wilcoxon rank-sum test, *P* > 0.05, Additional file 1: Table S5). Comparisons further revealed that children were enriched in bacteria from the phylum Bacteroidetes including *Bacteroides* and *Prevotella*, while Firmicutes assigned to the genera *Eubacterium*, *Clostridium*, *Dorea*, and *Coprococcus* were more abundant in adults (Additional file 2: Figure S1a) (adjusted *P* < 0.05, Fig. 1e). A recent large gut microbiome cohort study on 1135 Dutch adult participants also reported higher abundances of Firmicutes (63.7%) than Bacteroidetes (8.1%) [21]. By contrast, findings from a USA cohort revealed greater abundances of Firmicutes and significantly lower abundances of Bacteroidetes in healthy children aged 7–12 years than in healthy adults [17], indicating that the composition and development of bacterial communities varies in populations with different geographic and genetic origins.

At the functional level, a total of 6771 KEGG (Kyoto Encyclopedia of Genes and Genomes) orthologues (KOs) were identified in the childhood samples (median of 4695 per individual) and assigned to eggNOG (evolutionary genealogy of genes: Non-supervised Orthologous Groups) functional categories. An adult-like stable composition based on 25 eggNOG functional categories was observed in children (Additional file 2: Figure S1b).

In order to determine if the previously reported relation between bacterial gene richness and BMI in adults [22] was also observed in children, we examined whether bacterial gene richness differed between overweight children (BMI z-score ≥ 1.04, *n* = 23) and lean children (BMI z-score < 1.04, *n* = 258). A BMI z-score ≥ 1.04 was used as the threshold for identification of overweight in children as recommended by the Dutch National Growth Study [23]. Interestingly, we observed a bimodal distribution of bacterial gene counts in the overweight group (Additional file 3: Figure S2) where the children with a gene number < 600,000 (*n* = 8) exhibited significantly higher BMI z-score (Wilcoxon rank-sum test, *P* = 0.016). By contrast, no such gene distribution pattern was observed in lean children. We did not identify significant differences in relative species abundances between overweight and lean children (Wilcoxon rank-sum test, adjusted *P* > 0.05).

### Stratification of Dutch children based on their gut microbiome

Although, the concept of gut microbiota enterotyping has been highly debated [24], a consensus concerning enterotypes, including guidelines for rational enterotyping, was recently achieved [25]. Previous studies have suggested that human adult fecal metagenomes can be stratified into enterotypes that are independent of nationality, health status, age, BMI, or sex [26, 27], but associate strongly with long-term dietary patterns [28]. To examine this phenomenon in children, we here conducted a Dirichlet multinomial mixtures (DMM)-based enterotype analysis [29] to investigate the presence and characteristics of enterotypes in children based on the underlying compositional structure of their gut microbiome (see details in “Methods” section).

**Fig. 2:**
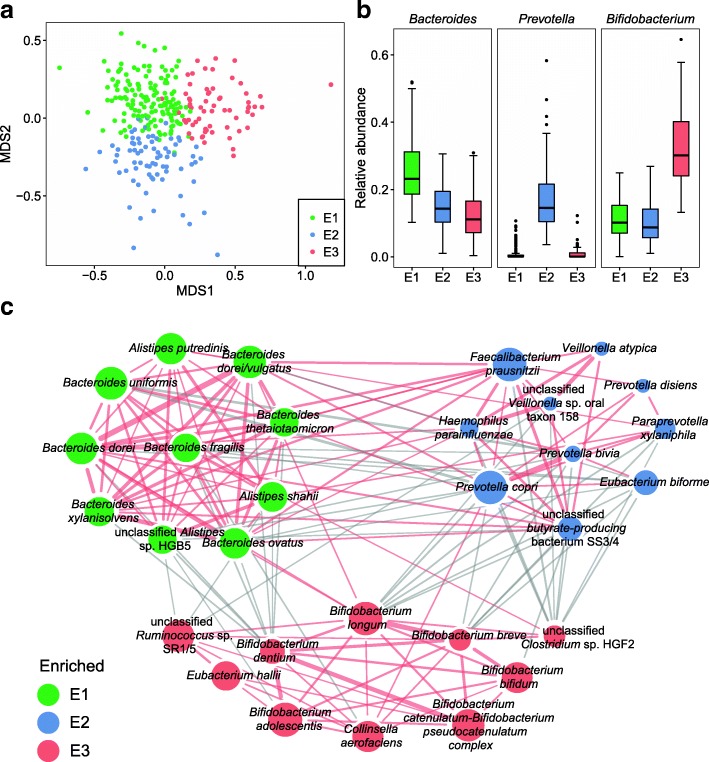
Stratification of early school-age children into three enterotypes based on their gut microbiome. **a** Scatter plot representing the three enterotypes identified using Dirichlet multinomial mixtures (DMM)-based clustering among early school-age Dutch children. *MDS* multidimensional scaling. **b** Genus abundance box plots showing the main contributors of each enterotype. **c** Correlations between enterotype enriched species, with the log10-transformed relative abundance of each species indicated by the circle area. Only the top 10 most abundantly enriched species in each enterotype are displayed. Red line indicates positive correlation and gray line indicates negative correlation (SparCC, pseudo *P* < 0.01)

Three enterotypes were identified and found to be driven by a relatively high abundance of the genera *Bacteroides* (enterotype 1 (E1), *n* = 143), *Prevotella* (enterotype 2 (E2), *n* = 74), and *Bifidobacterium* (enterotype 3 (E3), *n* = 64), respectively (Fig. 2a, b, Additional file 1: Table S6). Surprisingly, the abundances of 50 out of 81 detected genera and 132 out of 214 detected species (species with ≥ 100 detected genes in any enterotype) differed significantly between enterotypes (Kruskal-Wallis test, adjusted *P* < 0.05, Additional file 1: Tables S7, S8). All *Bifidobacterium* spp. exhibited higher abundances in enterotype 3 (E3), with the adult-type species *B*. *adolescentis* and *B*. *catenulatum* dominating this genus (58.8% of the genus) (Additional file 1: Table S8). SparCC analysis revealed strong positive correlations between species enriched within enterotypes while negative correlations were found between species enriched in different enterotypes (Fig. 2c). For instance, the relative abundance of *Prevotella copri* (E2) negatively correlated with both the abundance of *Bacteroides uniformis* (E1) and *Bifidobacterium longum* (E3) (SparCC, pseudo *P* < 0.01, Fig. 2c).

Comparative analyses further revealed distinct compositional and functional differences of the gut microbiota between the three enterotypes. Interestingly, children with a gut microbiota belonging to E1 and E2 showed similar gene count and diversity (*Dunn’s* post-hoc test, *P* > 0.05) while children with E3 harbored about 110,000 (15%) fewer genes (median value: 604,692 genes/child, Dunn’s post-hoc test, *P* < 0.05) compared to the children with the other two enterotypes (Additional file 1: Table S6, Additional file 3: Figure S2a). Kruskal-Wallis tests on differences between the numbers of genes in each genus further demonstrated that E3 harbored significantly fewer genes from unannotated bacteria and several abundant genera including *Bacteroides*, butyrate-producing *Faecalibacterium*, *Eubacterium*, and *Roseburia* (Additional file 1: Table S9). E3 also exhibited lower Shannon diversity (Dunn’s post-hoc test, *P* < 0.05, Additional file 4: Figure S3b), and higher reads mapping rates (median of 64.2%) to taxonomically annotated genes than the other two enterotypes (Dunn’s post-hoc test, *P* < 0.05, Additional file 4: Figure S3c). At the functional level, E3 showed a slightly lower number of KOs than E1 (Dunn’s post-hoc test, *P* < 0.05, Additional file 4: Figure S3d). However, compared to the other enterotypes, E3 displayed similar functional diversity (Dunn’s post-hoc test, *P* > 0.05, Additional file 4: Figure S3e) and higher reads mapping ratios to the KO annotated genes (Dunn’s post-hoc test, *P* < 0.05, Additional file 4: Figure S3f). Lower β diversity was identified between E1 and adults, as compared to that of adults vs. E2 or E3 (Kruskal-Wallis test, *P* < 0.05, Additional file 4: Figure S3g), overall suggesting that the gut microbiota development may continue beyond 6–9 years of age. Altogether, our data points to a more adult-like gut microbiome in children belonging to E1 and much simpler structured gut microbiome in children belonging to E3.

KEGG pathway enrichment analysis revealed distinct differences in microbial functional patterns between enterotypes. Metabolic modules involved in biosynthetic pathways for leucine, lysine, serine, methionine, proline, and histidine production, together with amino acid transport systems for glutamate, branched-chain amino acid (BCAA), and d-methionine, were highly enriched in the microbiome of E3 (Reporter score > 1.96, Additional file 5: Figure S4a). Conversely, the module for cysteine biosynthesis was less represented in E3 (Reporter score < − 1.96, Additional file 5: Figure S4a), in agreement with prior studies reporting the lack of cysteine synthetase in the genomes of *Bifidobacterium* species [30, 31]. Furthermore, compared to the other two enterotypes, the gut microbiota of children belonging to E3 showed higher enrichment of functions involved in metabolism of simple sugars including glycolysis and the pentose-phosphate pathway, while functions for utilizing complex carbohydrates such as pectin, uronic acids, and glycosaminoglycan degradation were depleted (Additional file 1: Table S10, Additional file 5: Figure S4a). Consistently, *Bacteroides* and *Prevotella* have been reported to possess large numbers of genes for fermentation and utilization of complex polysaccharides [32]. In contrast, in vitro culture experiments reported that *Bifidobacterium* strains grew well on glucose or ribose containing media but exhibited poor or no growth on media containing non-HMO-derived complex carbohydrates such as inulin or exopolysaccharide [33, 34]. Besides these differences, we also observed enterotype-dependent differences in the potential for biosynthesis of several water and lipid-soluble vitamins. For instance, children with a gut microbiota belonging to E1 showed higher potential for biosynthesis of cobalamin (B12) and biotin (B7), whereas children belonging to E2 exhibited higher potential for biosynthesis of menaquinone (vitamin K), pantothenate (B5), and riboflavin (B2) (Additional file 5: Figure S4a). A list of KEGG modules that differed significantly in abundance between enterotypes is provided in Additional file 1: Table S10. A heatmap showed that the relative abundance profiles of eight selected KOs, which are responsible for key steps in amino acid biosynthesis and carbohydrate metabolism, allowed to distinguish children in E3 from the two other groups (Additional file 5: Figure S4b).

### Associations between early events and pre-school lifestyle and gut microbiota in school-age children

**Fig. 3:**
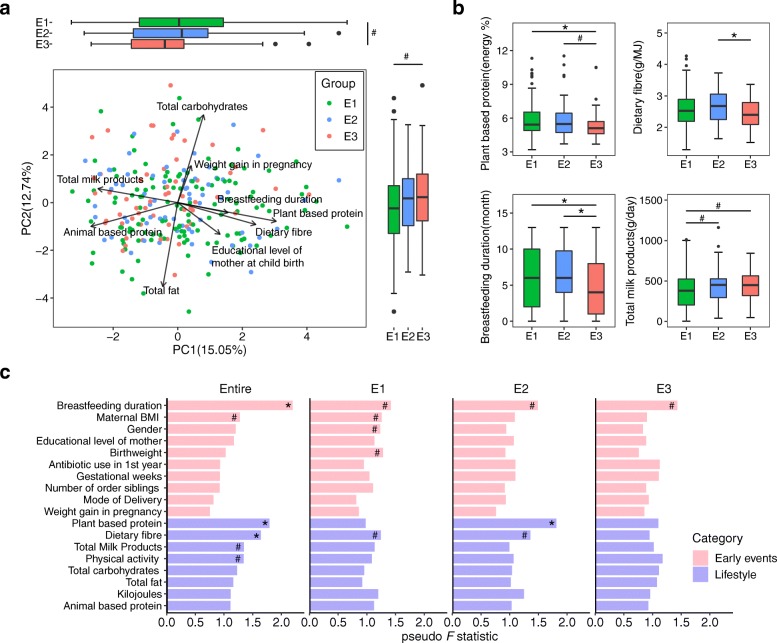
Multiple early events and pre-school lifestyle associated with the school-age gut microbiota. **a** PCA showing the multivariate variation of children and the major contributions of different factors to PC1 and PC2. A total of 18 factors including early events and pre-school lifestyle (Additional file 1: Table S1) were subjected to PCA, and those factors with component scores for PC1 or PC2 ≥ 0.2 were shown as major contributors. Box plots showing the overall distribution of PC1 and PC2 scores within each enterotype (#*P*<0.05; Wilcoxon rank-sum test). **b** Significant major contributors in PCA between enterotypes (#*P* < 0.05, Wilcoxon rank-sum test; *P < 0.05, Dunn’s post-hoc test). The details of statistical results for all factors are shown in Additional file 1: Table S11. **c** PERMANOVA of the influence of single-factor early events and pre-school lifestyle on the gut microbial gene profile in the entire cohort and within each enterotype (#*P* < 0.05; * adjusted *P* < 0.05)

In order to identify interactions between early environmental factors and the microbiota, we first conducted a PCA to assess the multivariate variation in children of the early events, pre-school dietary, and non-dietary lifestyle factors (Additional file 1: Table S1). We found that breastfeeding duration, educational level of mother at childbirth, and pre-school dietary patterns including intake of protein, fiber, and milk products contributed most to the variability in PC1 (15.05%, Fig. 3a), and total intake of carbohydrates and fat represented the second most important variation among children, as displayed in PC2 (12.74%, Fig. 3a). Interestingly, children in E3 exhibited lower PC1 scores but higher PC2 scores than children in E1 (Fig. 3a, Wilcoxon rank-sum test, *P* < 0.05). This inter-enterotype difference was governed by specific major contributors of the PC1 scores, including shorter breastfeeding duration and less intake of dietary fiber and plant-based protein in E3 as compared to the two other enterotypes (Kruskal-Wallis test, *P* < 0.05) (Fig. 3b, Additional file 1: Table S11). Next, we performed permutational multivariate analysis of variance (PERMANOVA) to assess the interactions between early events, pre-school lifestyle, and microbial gene profiles among all individuals and then went on to evaluate their interactions in each enterotype. Based on the entire population of children, early events including breastfeeding duration, the pre-school lifestyle including intake of plant-based protein and dietary fiber were significantly correlated with the microbial composition at 6–9 years of age (adjusted *P* < 0.05, Bray-Curtis distance, Fig. 3c). In addition, we identified several different correlation patterns within each enterotype (Fig. 3c). For instance, plant-based protein intake was significantly correlated with the gut microbiota in E2 (adjusted *P* < 0.05), but not in E1 and E3 (adjusted *P* > 0.05).

Spearman’s rank correlation analyses further revealed significant correlations between early events, pre-school lifestyle, and the relative abundance of certain microbial species (Additional file 6: Figure S5a, adjusted *P* < 0.05). Interestingly, bacteria enriched in E3 such as *B*. *adolescentis*, *B. angulatum*, and *B*. *breve* were negatively correlated with breastfeeding duration (Additional file 6: Figure S5a). The relative abundances of several *Streptococcus* species, reported to be part of the oral cavity microbiota of children [35–37], were positively related to maternal BMI (Additional file 6: Figure S5a, adjusted *P* < 0.05). The relative abundances of *B*. *angulatum*, *B*. *dentium*, and *Streptococcus mitis* were negatively correlated with plant-based protein intake (adjusted *P* < 0.05). Moreover, several species showed correlations with both early events and pre-school dietary patterns before age 5. For instance, *Streptococcus parasanguinis* and *Streptococcus gordonii* that associated with maternal BMI were also positively correlated with animal-based protein intake (adjusted *P* < 0.05). Similarly, the relative abundances of *Collinsella intestinalis* showed a positive correlation with maternal BMI and a negative correlation with dietary fiber intake (adjusted *P* < 0.05). Collectively, our findings indicate that both early events and pre-school dietary lifestyle contribute to shaping of the gut microbiota in school-age Dutch children, with different factors influencing each enterotype.

### Enterotype-dependent associations between school-age metabolic phenotypes and pre-school lifestyle

An increasing number of studies has linked the gut microbiota with host metabolic phenotypes, including glucose and insulin homeostasis, and amino acid metabolism [38–40]. We examined the association between the gut microbiota and metabolic phenotypes including glucose, insulin, and amino acids levels measured in blood samples collected 3.5 h after the last meal on the same day as the fecal samples were collected (Additional file 6: Figure S5b). We observed that the relative abundances of *Bacteroides* spp., including *Bacteroides xylanisolvens*, *B*. *dorei*, *B*. *vulgatus*, and *B*. *eggerthii*, enriched in E1, were negatively correlated with plasma branched-chain amino acid (BCAA) levels. It has been demonstrated in a single mouse study that certain *Bacteroides* strains may contribute to BCAA degradation and hence reduce the circulating levels of BCAA in the host [41]. In addition, we also observed positive correlations between *Dorea longicatena*, *Coprococcus comes*, and plasma glutamate levels, which is in line with a recent study reporting an enrichment of these species and plasma glutamate levels in young obese Chinese adults [42]. The abundance of *S*. *gordonii* positively correlated with plasma total triglyceride and glucose.

Next, we examined if the measured levels of blood metabolic parameters, such as insulin and glucose, in school-age children differed between the three enterotypes. No differences were observed between individuals within the three enterotypes (Additional file 1: Table S11, Kruskal-Wallis test, *P* > 0.05).

Given the multi-relationships between gut microbiota and the above-mentioned early events and pre-school lifestyle, we next asked whether these factors would impact metabolic responses in the early school-age children. We first conducted Spearman’s rank correlation analyses between early factors and school-age metabolic phenotypes across the entire children cohort. We found that insulin levels exhibited negative associations with the pre-school lifestyle related to intake of plant-based protein and dietary fiber (Spearman’s correlation, adjusted *P* < 0.05, Additional file 7: Figure S6a). However, by stratifying according to enterotypes, we observed different correlation patterns among children within the different enterotypes. For instance, only children within E1 and E2 exhibited negative associations between insulin levels and intake of plant-based protein and dietary fiber (adjusted *P* < 0.05, Additional file 7: Figure S6b, c). In addition, the negative correlations between increased pre-school dietary fiber intake and reduced total serum triglyceride (TG) levels at school-age were solely present in E2 (Additional file 7: Figure S6c). Despite no such relationships were seen for E3 children, we found plasma free fatty acid (FFA) levels positively correlated with animal-based protein (Additional file 7: Figure S6d), and additionally, a few *Streptococcus* spp. to be positively correlated with the plasma FFAs levels only in this group (Additional file 8: Figure S7). Most of these enterotype-dependent associations persisted after adjusting for multiple covariates including sex, age, and BMI z-score at stool collection and early events by linear regression models (Additional file 1: Table S12).

**Fig. 4:**
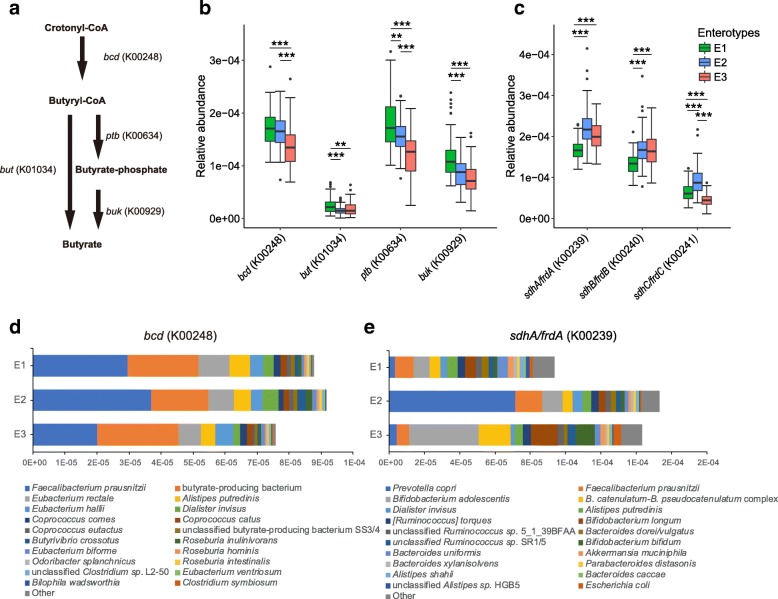
Enterotype-associated differences in potential for butyrate, succinate, and propionate production. **a** Pathway showing the genes involved in final biosynthetic steps from crotonyl-CoA to butyrate, including *bcd* (butyryl-CoA dehydrogenase, K00248), *ptb* genes (phosphate butyryltransferase, K00634), *buk* genes (butyrate kinase, K00929), and *but* (butyryl CoA:acetate CoA transferase, K01034). **b** The relative abundance of genes involved in butyrate production within each enterotype. **c** The relative abundance of genes involved in succinate production (succinate dehydrogenase complex (K00239, K00240 and K00241) within each enterotype. **d** Mean relative abundance of *bcd* genes (K00248) listed according to annotated bacterial species within each enterotypes. **e** Mean relative abundance of *sdhA* genes (K00239) listed according to annotated bacterial species within each enterotypes. Dunn’s post-hoc test; **P < 0.01, ***P < 0.001

Further, the children in E3 who did not exhibit a negative correlation between pre-school dietary lifestyle of plant-based protein and dietary fiber intake and blood insulin levels at school-age exhibited a lower potential for complex carbohydrate metabolism (Additional file 5: Figure S4, Additional file 7: Figure S6). We assumed that the different responses of insulin levels to dietary fiber intake might be due to variations in metabolites reported to influence systemic insulin levels such as butyrate [13] generated by the gut microbiota in response to intake and fermentation of complex carbohydrates. Hence, we compared the abundances of genes involved in the conversion of crotonyl-CoA to butyrate, which is one of the final steps for butyrate production. Interestingly, the abundance of the *bcd* gene encoding butyryl-CoA dehydrogenase (K00248) was significantly enriched in both E1 and E2 as compared to E3. Moreover, the abundances of *ptb* (phosphate butyryltransferase, K00634), *buk* (butyrate kinase, K00929), and *but* (butyryl CoA: acetate CoA transferase, K01034) were all significantly enriched in E1 as compared to E2 and E3, suggesting a higher potential for butyrate production in E1 (Fig. 4a, b). A taxa distribution analysis revealed that *Faecalibacterium prausnitzii*, *Eubacterium halii*, *Roseburia inulinivorans*, and *Odoribacter splanchnicus* largely accounted for the prevalence of the *bcd* genes when focusing on differences of butyrate production potentials between enterotypes (Fig. 4d, Additional file 1: Table S13). We further compared the relative abundance of genes required for succinate production between the three enterotypes, since succinate has been reported as a microbial product produced in response to dietary fiber intake that may contribute to improved plasma glucose and body weight regulation [43]. As shown, E2 exhibited significantly higher abundances of genes encoding the succinate dehydrogenase complex (K00239, K00240, and K00241) than E1, and the dominant contributor in E2 was *Prevotella copri* (Fig. 4c, e). Despite the fact that propionate is commonly converted from the succinate pathway in the intestine [44], no differences were observed between the three enterotypes with respect to the relative abundance of *pct* genes (K01026) encoding the propionate CoA-transferase, which is responsible for the last step in propionate production (Additional file 1: Table S14, Kruskal-Wallis test, *P* > 0.5).

Collectively, our results illustrate that early events and pre-school dietary lifestyle impact on the development of the gut microbiota, which in turn seems to affect host metabolic responses.

## Discussion

Comprehensive information on the structural and functional configuration of the gut microbiota of early school-age children is limited. Previous studies have surveyed this age-specific population using 16S rRNA gene amplicon sequencing or metagenomic shotgun sequencing with relatively small sample sizes [17, 18]. In this study, we provide a comprehensive overview of the gut microbial characteristics of 281 early school-age Dutch children, displaying an enrichment of bacteria from the Bacteroidetes and Actinobacteria phyla, and a similar functional composition as compared to Dutch healthy adults. Furthermore, we identified three enterotypes in healthy children, with children dominated by *Bifidobacterium* showing the lowest gene number and the lowest diversity compared to children enriched by *Bacteroides* or *Prevotella*, suggesting stratified developmental trends of the childhood gut microbiota toward an adult-like configuration.

Further supporting the concept of the importance of environmental factors in shaping the development of the gut microbiota, we found several factors, especially breastfeeding duration in early life and plant-based food intake (dietary fiber and plant-based protein) in pre-school children (adjusted *P* < 0.05), to significantly correlate with the gut microbiota composition in school-age children. Moreover, we identified a shorter breast-feeding duration in the *Bifidobacterium*-dominated enterotype (E3) in school-age children than in other groups. This was reflected in significant negative correlations between a few *Bifidobacterium* species and breastfeeding duration including the most abundant adult-type *B. adolescentis*. In pre-school children, less than average dietary fiber intake was also observed in E3, in accordance with previous studies that reported significantly lower abundance of *Bifidobacterium* in healthy subjects on fiber-blend formula than subjects on fiber-free formula [45], and lower *Bifidobacterium* in vegans than in vegetarians [46]. Functional analyses demonstrated that children with a gut microbiota enriched in *Bifidobacterium* strains favored utilization of simple sugars but lacked the potential for complex carbohydrate utilization. In addition, a recent Dutch adult cohort study reported that the relative abundance of *Bifidobacterium* in the gut could be affected by an interaction between genotype and intake of dairy products, with adults of the GG genotype on rs4988235 in the *MCM6* gene showing a positive relationship between *Bifidobacterium* abundance and milk product consumption [16]. Combined, the present and previous findings suggest that intestinal *Bifidobacterium* levels might not only be influenced by early events and dietary patterns but also by host genetics. However, the detailed mechanisms underlying colonization and establishment of *Bifidobacterium* species in the human gastrointestinal tract remain to be determined.

Dietary patterns have been shown to substantially impact on the gut microbiota and host metabolism. A key finding of this study is the enterotype-stratified association pattern between school-age metabolic phenotypes and pre-school dietary patterns. Although the benefits of a high-fiber diet have been well documented in epidemiological studies, we only observed significant negative correlations between plant-based diet intake (dietary fiber and plant-based protein) and insulin levels in children with high abundances of *Bacteroides* or *Prevotella*, but not in children with high *Bifidobacterium* abundance. Similarly, the dietary fiber-induced improvement in postprandial glucose response has also been shown to be associated with increased abundance of *Prevotella* in a recent human intervention study [48]. In our search for possible functional explanations for the identified enterotype-dependent correlations, we discovered very distinct gut microbial functional capacities between enterotypes, particularly in relation to the potential for transformation of metabolites derived from microbial fermentation. Thus, the functional potential for fermentation of complex carbohydrate was less in children with high *Bifidobacterium* abundance compared to others. Focusing on the two groups with higher fermentation potential, we observed that children with a *Bacteroides*-driven enterotype exhibited a higher abundance of a set of genes (*buk* and *but*) related to butyrate biosynthesis, while children with a *Prevotella*-driven enterotype possessed higher potential for succinate production. Both butyrate and succinate have been demonstrated to exert beneficial effects in relation to glucose and insulin homeostasis [13, 43]. Together, our findings support the notion that host metabolic benefits of plant-based diets might be conferred by specific bacteria and specific metabolites derived from carbohydrate fermentation. In addition, several *Streptococcus* spp. exhibited negative correlations with dietary fiber and plant-based protein intake in E2, but not in E1 and E3. *Streptococcus* spp. being high in E3 might also play a role in the enterotype-based metabolic responses to dietary patterns in this group due to the reported positive correlation with total fat consumption and elevated plasma free fatty acid levels. Further well-controlled dietary intervention studies are warranted to confirm the metabolic responses to dietary factors and establish the causal roles for these bacterial taxa and functional pathways in determining the host metabolic benefits observed in this study.

In conclusion, this study reveals important characteristics of the gut microbial structure and function in healthy early school-age Dutch children, which are affected by certain early life factors, such as breastfeeding duration and pre-school dietary habits, while the influence from physical activity is negligible. Of specific note, our findings suggest that distinct metabolic responses to dietary lifestyle are strongly governed by the composition and functional potentials of the gut microbiota, implying that stratification of children according to gut microbiota enterotypes may well be included in future investigations on the relationship between dietary intake and metabolic health in children.

## Method

### KOALA study population

The 281 Dutch children were part of the KOALA Birth Cohort Study. The design of the study has been described in detail elsewhere [47]. Inclusion criteria for the present study were the availability of fecal and blood samples collected in children 6 to 9 years of age (72–108 months). Children born prematurely, twins, children with abnormalities linked to growth (such as Down’s syndrome, Turner syndrome, Fallot’s tetralogy, multiple disabilities, and cystic fibrosis), and children treated with antibiotics within 4 weeks prior to fecal sample collection were excluded. Fecal samples were collected as previously described [48]. Blood samples and anthropometric measures were collected during home visits within the week of fecal collection. Height and weight were measured by trained research assistants. BMI measurements were converted into age- and gender-specific z-scores using data from children enrolled in the Dutch National Growth Study as the reference population and converted into dichotomous outcomes: lean vs. overweight (BMI z-score > 1.04, corresponding to the 85th percentile according to standard guidelines [23]).

Blood samples were collected approximately 3.5 h after the last meal intake. Data on early events were collected through repeated parent-reported questionnaires during pregnancy and early life. Lifestyle characteristic including dietary intake (Food Frequency Questionnaire) and physical activity were collected through questionnaires at 4–5 years as described previously [49]. Blood biochemical indices were measured by radioimmunoassay (insulin, adiponectin, leptin) or with enzymatic/colorimetric methods on an automated spectrophotometric analyzer (free fatty acids, glucose, total and HDL cholesterol, triglycerides, high-sensitivity C-reactive protein). Plasma amino acids were measured by ultra-performance liquid chromatography–tandem mass spectrometry (UPLC-MS/MS) [50]. The summary of sample information is presented in Additional file 1: Table S1.

### Generation of children fecal metagenomic data

Libraries with an insert size of 350 base pair (bp) were constructed from metagenomic DNA for each sample following the manufacturer’s instructions (Illumina, San Diego, California, USA). Illumina sequencing with 100 bp paired-end reads was applied to all 281 samples.

### Availability of public Dutch adult metagenomic data

Illumina-based paired-end metagenomic sequencing data were collected from 62 healthy Dutch adult controls from the Maastricht IBS cohort (MIBS-CO) [16]. The sample information is presented in Additional file 1: Table S3.

### Profiling of metagenomic samples

The raw sequencing data from the KOALA cohort were processed for quality control using the FASTX toolkit in the MOCAT pipeline [51]. We trimmed reads with continuous bases from the 3′-end of a read with average Phred score ≤ 20 and kept the remaining high-quality reads with length larger than 30 bp. The high-quality reads were then aligned to hg19 using SOAP2.2 (identity ≥ 0.9) to remove human reads. The high-quality non-human reads were defined as clean reads and aligned against the human gut microbial integrated gene catalog (IGC) to generate count profiles using SOAP2.2 (identity ≥0.95) [15].

To eliminate the influence of sequencing depth in comparative analyses, unique IGC mapped reads of each sample were downsized to 20 million for each child. After this, we identified 268,363 to 1,069,059 microbial genes in the 281 samples, with an average of 694,404 genes per sample. The relative abundance profiles of genes, genera, species, and KOs were determined by summing the relative abundance of genes from each taxon or KO using the downsized mapped reads per sample [15]. Due to the relatively limited amount of data (average 13.5 million uniquely mapped reads per adult), the metagenomic datasets from Dutch adults were processed to calculate Shannon index and generate gene, genus, KO, and eggNOG profiles using the same pipeline as described above without reads downsizing. Data statistics for each sample are provided in Additional file 1: Table S2.

### Richness, diversity, and enterotype analysis

Alpha diversity (within-sample diversity) was quantified by the Shannon index using gene relative abundance profiles [15]. Beta diversity (between-sample diversity) was calculated using Bray-Curtis distance (R 3.2.5, *vegan* package 2.4-4). For Dutch children, the gene and KO counts which represented gene or functional richness were calculated in each downsized sample in accordance with the previous study [22].

Genus-level enterotypes analysis was performed according to the Dirichlet multinomial mixtures (DMM) and partitioning around medoid (PAM)-based clustering protocols using Jensen-Shannon divergence (PAM-JSD) and Bray-Curtis (PAM-BC) (Additional file 9: Figure S8) [29, 27]. The optimal cluster number of the protocols was calculated using Laplace approximation and Calinski–Harabasz index [29, 27], with all protocols indicating an optimal cluster number of three among the 281 Dutch children (Additional file 9: Figure S8a–c). We next randomly selected 200, 220, 240, 260, and 280 samples for enterotype clustering to evaluate clustering stability of the three protocols, by repeating the above sampling and clustering process 10 times for each sample size and generating enterotyping results of 50 times for each method. The DMM-based protocol, which showed a higher consistency than the two PAM-based protocols, was chose for this study (Additional file 9: Figure S8d–f).

### KEGG enrichment analysis

Differentially enriched KEGG modules were identified according to reporter Z-scores. One-tail Wilcoxon rank-sum test was performed on all the KOs that occurred in more than six samples and adjusted for multiple testing using the Benjamini-Hochberg method [52]. The Z-score for each KO was calculated based on the adjusted *P* value for a particular KO and the aggregated Z-score for each module was calculated from the Z-scores of all KOs involved in the module [6]. An absolute value of reporter score of 1.96 (95% confidence according to normal distribution) or higher was used as the detection threshold for significance.

### Statistical analyses

Before comparison, the taxa with low coverage of annotated gene number (less than 100) in the entire cohort were filtered for further analyses and this confined our analysis to 82 genera and 226 species. Principal component analysis (PCA) was implemented using the function *prcomp* in R 3.2.5.

Wilcoxon rank-sum tests were conducted to detect differences in the gut microbial characteristics, including gene count, Shannon index, and the relative abundances of genera, species, and KOs between the Dutch children and adults, and between overweight and lean children. Kruskal-Wallis tests were performed to assess the differences in gut microbial characteristics and continuous phenotypic variables between enterotypes. Dunn’s post-hoc tests followed by pairwise comparisons were performed to explore the differences between two groups. To detect the differences in categorical phenotypic variables, Chi-square tests were conducted. The Benjamini-Hochberg method was used for multiple testing correction [52], with cutoff for adjusted *P* value at 0.05.

Permutational multivariate analysis of variance (PERMANOVA) was performed to assess the correlation between gene-level microbial profiles and phenotypic factors including early events and lifestyle (Bray-Curtis distance). The pseudo *F* statistics and *P* values were calculated using the function *adonis* from vegan package in R 3.2.5 based on 9999 permutations. The cutoff was set as adjusted *P* < 0.05.

SparCC was run with default parameters and 1000 bootstraps to test for correlations between the relative abundances of species [53]. Pseudo *P* values were calculated as the proportion of simulated bootstrapped data sets with a correlation at least as extreme as the one computed for the original data set. The significant cutoff for SparCC was set at pseudo *P* < 0.01. The co-occurrence network of species was visualized in Cytoscape 3.5.1.

Spearman’s rank coefficient correlation (SCC) analysis was used for correlations between continuous phenotypic factors and between continuous phenotypic factors and microbial species or KOs. The significant cutoff for SCC was set at an adjusted *P* < 0.05.

General linear model (GLM) regression analyses were conducted to validate the enterotype-dependent associations between pre-school lifestyle and school-age blood metabolic parameters determined by Spearman correlation analysis. Two confounder adjustment models were applied, with model 1 adjusting for gender, age, and BMI z-score and model 2 adjusting for gender, age, BMI z-score, and all early events. Shapiro-Wilk test was conducted to test for normality of residuals of the regression models. Box-Cox transformation was conducted on dependent variables of non-normally distributed residuals. Residuals were considered as normally distributed with *P* > 0.05 (Shapiro-Wilk test). The regression coefficient (β) and two-tailed *P* values for the coefficient were calculated (glm in R 3.2.5). The *P* < 0.05 was regarded as significant.

## Additional files

Additional file 1: **Table S1.** Summary of phenotypic information of Dutch children at 6–9 years of age. Table S2. Sequencing statistics for fecal samples of Dutch children. Table S3. Summary of phenotypic and sequencing information of Dutch adults. Table S4. List of genera that differ significantly in abundance between Dutch children and adults. Table S5. List of species that differ significantly in abundance between Dutch children and adults. Table S6. Dirichlet multinomial mixtures (DMM)-based enterotypes in Dutch children. Table S7. List of genera that differ significantly in abundance between enterotypes. Table S8. List of species that differ significantly in abundance between enterotypes. Table S9. Number of genes annotated at the genus level in enterotypes. Table S10. Differential enrichment of KEGG modules between enterotypes. Table S11. Differences in host phenotypic parameters between enterotypes. Table S12. Generalized linear analysis for the association between pre-school dietary factors and school-age blood metabolic parameters. Table S13. Taxa distributions of genes involved in butyrate and succinate production. Table S14. List of genes involved in butyrate, propionate and succinate production. (XLSX 241 kb)

Additional file 2: **Figure S1.** Compositional and functional comparison between Dutch children and adults. Relative abundance of major phyla in Dutch children (a) and adults (b). (c) Relative abundance of COG (clusters of orthologous groups) categories across each sample in Dutch children and adults. (PDF 298 kb)

Additional file 3: **Figure S2.** Gene count distribution in Dutch children. Black indicates all individuals, *n* = 281; red indicates lean children (BMI z-score < 1.04, *n* = 258) and blue indicates overweight children (BMI z-score ≥ 1.04,*n* = 23). A bimodal distribution of bacterial gene counts observed in the overweight group with the children having gene numbers lower than 600,000 (*n* = 8) showing significant higher BMI z-score (Wilcoxon rank-sum test, *P* = 0.016). (PDF 123 kb)

Additional file 4: **Figure S3.** Comparison of gut microbial compositional and functional structure between enterotypes. (a-c) Comparison of gene count, gene-based Shannon diversity and reads mapping ratio to the taxonomic annotated genes between enterotypes. (d-f) Comparison of gene count, KO-based Shannon diversity and reads mapping ratio to the KO annotated genes between enterotypes. *Dunn’s* post hoc test, *, *P*<0.05; **, *P* < 0.01; ***, *P* < 0.001. The fraction of reads mapped to genes with taxonomic or KO annotation was calculated by dividing the number of reads mapped to annotated genes by the total number of reads mapped to IGC. (g) Comparison of beta diversity between enterotype-based children and adults. (PDF 204 kb)

Additional file 5: **Figure S4.** Comparison of gut microbial functional potentials between enterotypes. (a) Differentially enrichment of KEGG modules between enterotypes. Dashed lines indicate a reporter score of 1.96, corresponding to 95% confidence in a normal distribution. (b) Heatmap showing that the relative abundance profiles of 8 selected KOs involved in key functions of metabolic pathways for carbohydrate metabolism (K00845, K01051 and K00873) and amino acid biosynthesis (K01738, K00928, K00058, K00651and K00765) distinguishes E3 from E1 and E2. (PDF 358 kb)

Additional file 6: **Figure S5.** Correlations between continuous phenotypic parameters and species profile in the entire cohort. (a) Spearman’s rank correlations between early events, pre-school lifestyle and species profile (n = 281). (b) Spearman’s rank correlations between blood parameters and species profiles (n = 281). *P* values were adjusted for each parameter. The “*” indicates significant correlation with adjusted *P* < 0.05. Species significantly correlated with at least one factor are presented. (PDF 301 kb)

Additional file 7: **Figure S6.** Correlations between continuous phenotypic parameters. (a) Spearman’s rank correlations between continuous phenotypic parameters in the entire cohort (n = 281). (b) Spearman’s correlations between continuous phenotypic parameters in E1 (*n* = 143). (c) Spearman’s rank correlations between continuous phenotypic parameters in E2 (*n* = 74). (d) Spearman’s rank correlations between continuous phenotypic parameters in E3 (*n* = 64). The “*” indicates significant correlation with adjusted *P* < 0.05 (PDF 296 kb)

Additional file 8: **Figure S7.** Correlations between *Streptococcus* species profile and selected phenotypic parameters in enterotypes. Heatmap showing the Spearman’s rank correlations between *Streptococcus* species and selected phenotypic parameters including free fatty acids levels and the intake of total carbohydrate, total fat, dietary fiber, and plant-based protein. P values were adjusted for each parameter. The “*” indicates significant correlation with adjusted *P* < 0.05. The “#” indicates correlation with *P* < 0.05 and adjusted *P* > 0.05. FFA, free fatty acids. (PDF 197 kb)

Additional file 9: **Figure S8.** Evaluation of enterotying protocols for Dutch children. (a-c) Evaluation of optimal cluster number by using the DMM protocol (a), the PAM-JSD protocol (b) and the PAM-BC protocol (c). The optimal number of clusters was calculated using Laplace approximation for the DMM protocol (a) and the Calinski–Harabasz index for the PAM-based protocols (b-c). Cluster stability using the DMM (d), the PAM-JSD (e) and the PAM-BC protocols (f). The X axis indicates resampling number and the Y axis indicates the consistency of resampling relative to the original result based on 281 samples. (PDF 221 kb)

## Abbreviations

BCAA: Branched-chain amino acid
DMM: Dirichlet multinomial mixtures
eggNOG: Evolutionary genealogy of genes: Non-supervised Orthologous Groups
GPCRs: G protein-coupled receptors
IGC: Integrated gene catalog
KEGG: Kyoto Encyclopedia of Genes and Genomes
PCA: Principal component analysis
PERMANOVA: Permutational multivariate analysis of variance.
SCFAs: Short-chain fatty acids
