# Supplementary figures and images for: Impact of early events and lifestyle on the gut microbiota and metabolic phenotypes in young school-age children
Source: Microbiome. 2019 Jan 4;7:2. doi: 10.1186/s40168-018-0608-z (PMC6320620; doi:10.1186/s40168-018-0608-z)

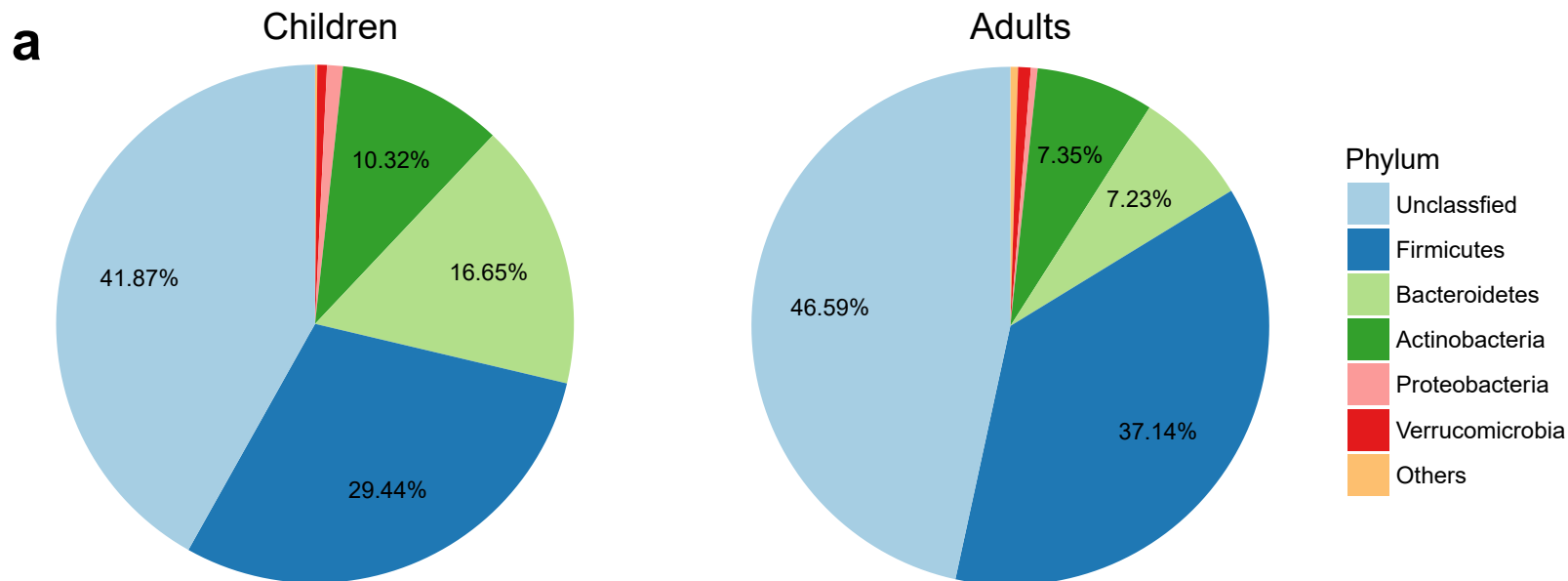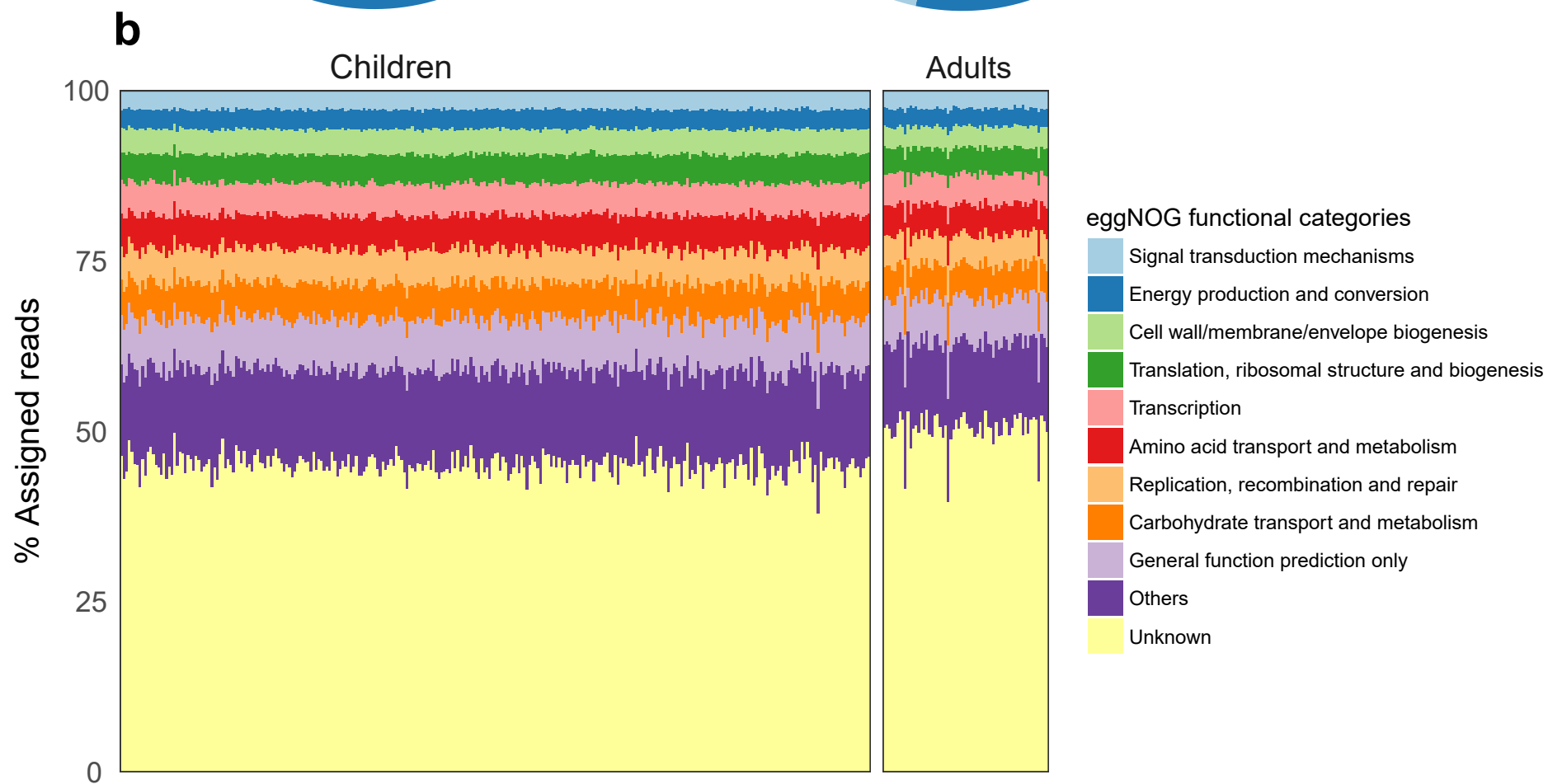

Supplement: Supplementary file 2 — Figure S1. Compositional and functional comparison between Dutch children and adults. Relative abundance of major phyla in Dutch children (a) and adults (b). (c) Relative abundance of COG (clusters of orthologous groups) categories across each sample in Dutch children and adults. (PDF 298 kb) [file 40168_2018_608_MOESM2_ESM.pdf]

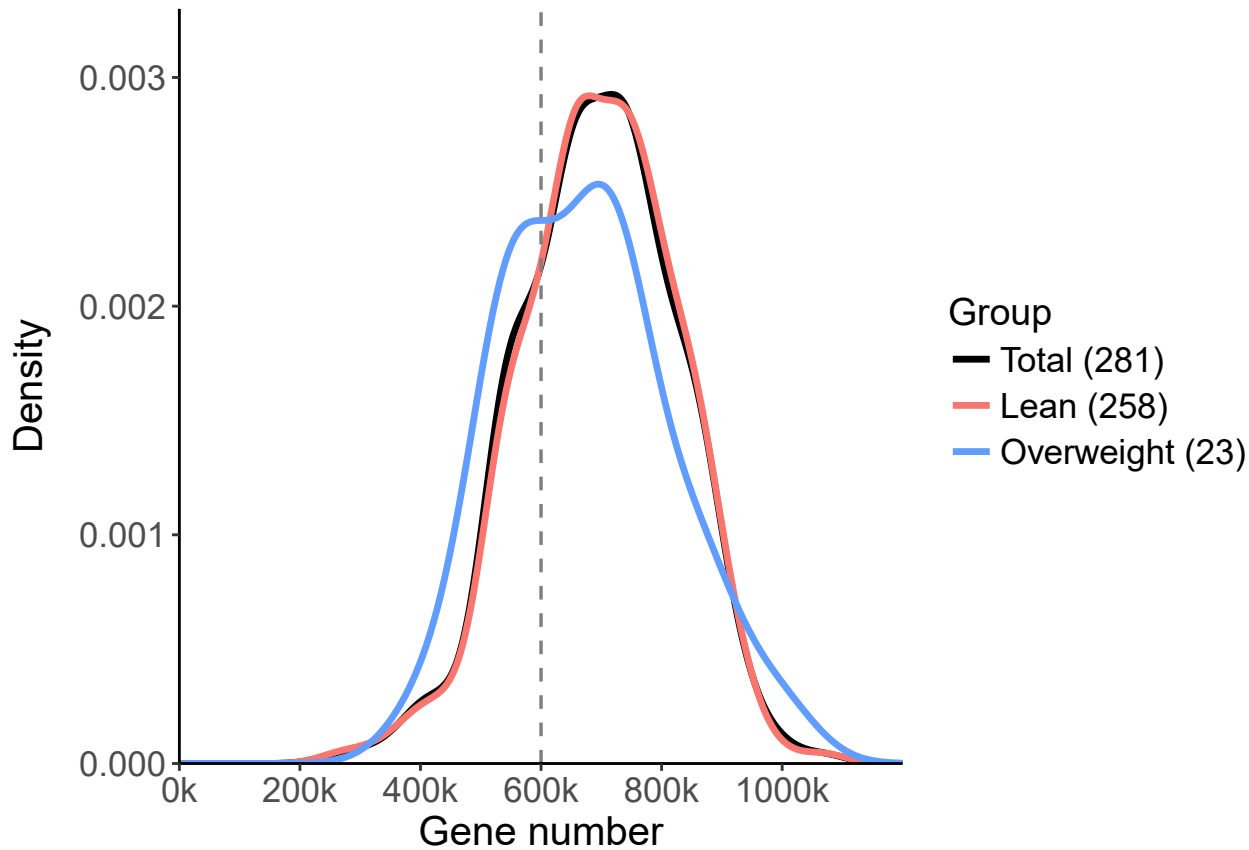

Supplement: Supplementary file 3 — Figure S2. Gene count distribution in Dutch children. Black indicates all individuals, n = 281; red indicates lean children (BMI z-score < 1.04, n = 258) and blue indicates overweight children (BMI z-score ≥ 1.04,n = 23). A bimodal distribution of bacterial gene counts observed in the overweight group with the children having gene numbers lower than 600,000 (n = 8) showing significant higher BMI z-score (Wilcoxon rank-sum test, P = 0.016). (PDF 123 kb) [file 40168_2018_608_MOESM3_ESM.pdf]

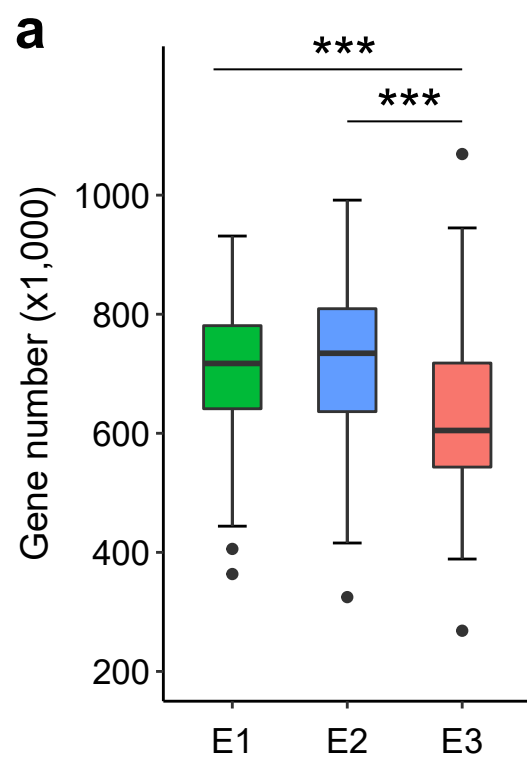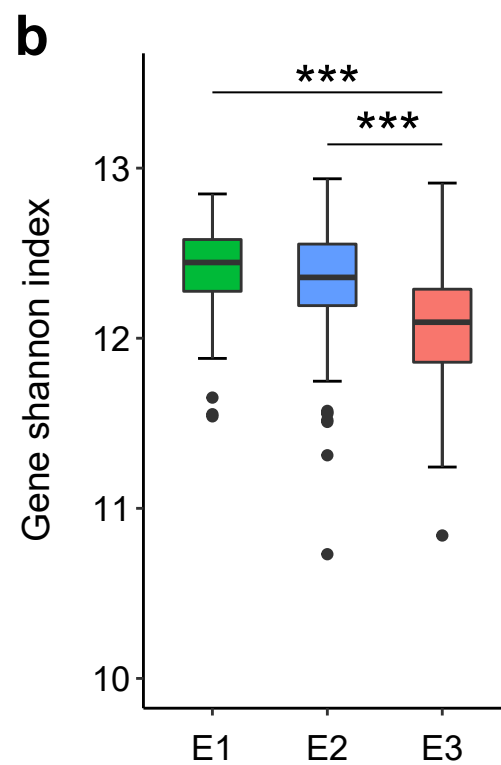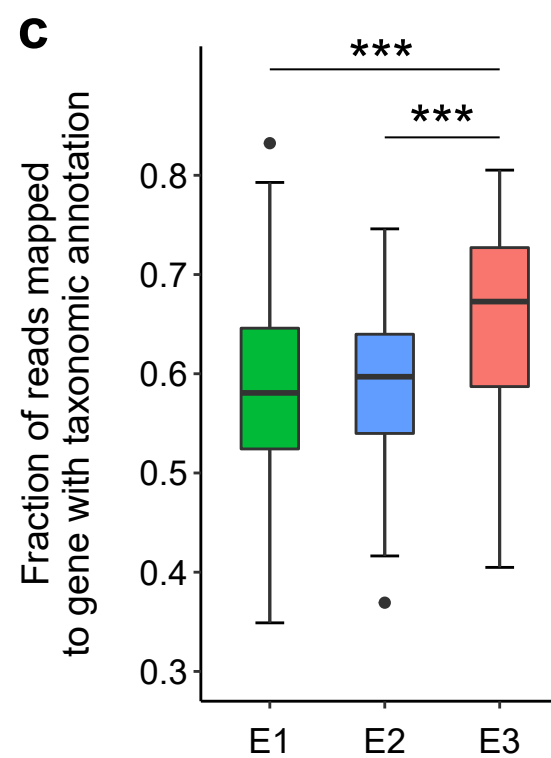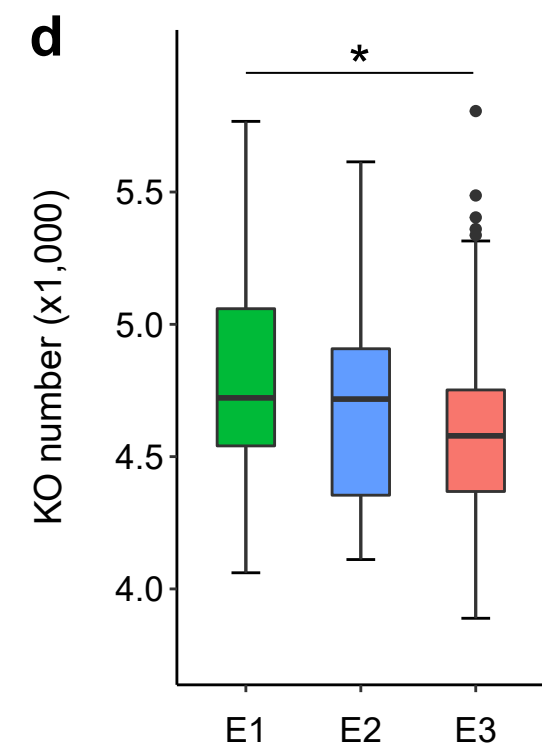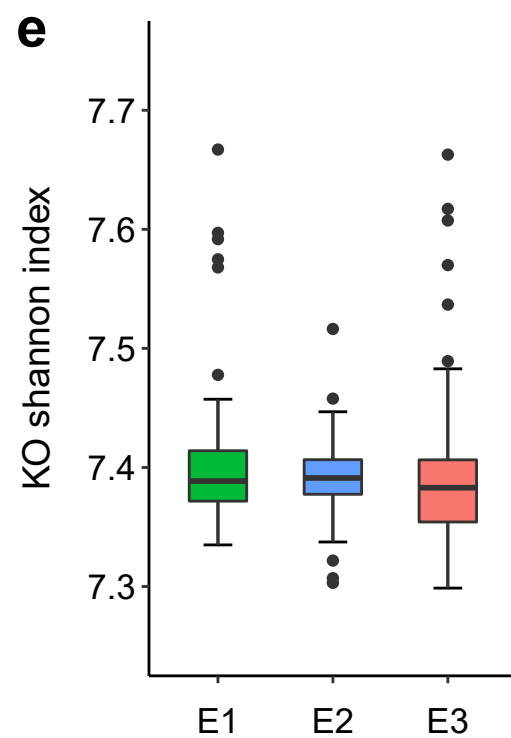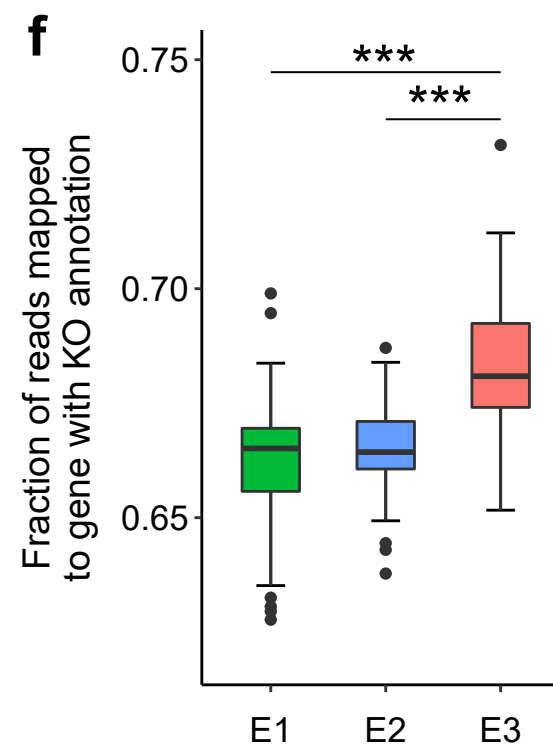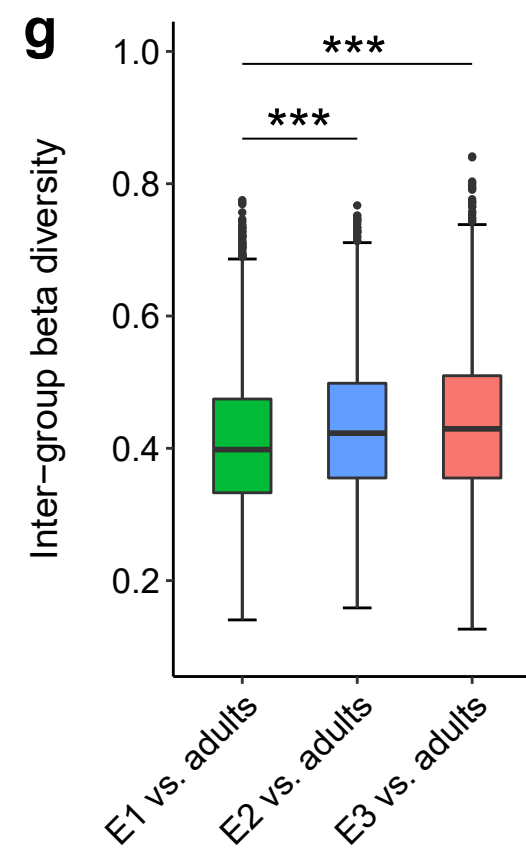

Supplement: Supplementary file 4 — Figure S3. Comparison of gut microbial compositional and functional structure between enterotypes. (a-c) Comparison of gene count, gene-based Shannon diversity and reads mapping ratio to the taxonomic annotated genes between enterotypes. (d-f) Comparison of gene count, KO-based Shannon diversity and reads mapping ratio to the KO annotated genes between enterotypes. Dunn’s post hoc test, *, P<0.05; **, P < 0.01; ***, P < 0.001. The fraction of reads mapped to genes with taxonomic or KO annotation was calculated by dividing the number of reads mapped to annotated genes by the total number of reads mapped to IGC. (g) Comparison of beta diversity between enterotype-based children and adults. (PDF 204 kb) [file 40168_2018_608_MOESM4_ESM.pdf]

a

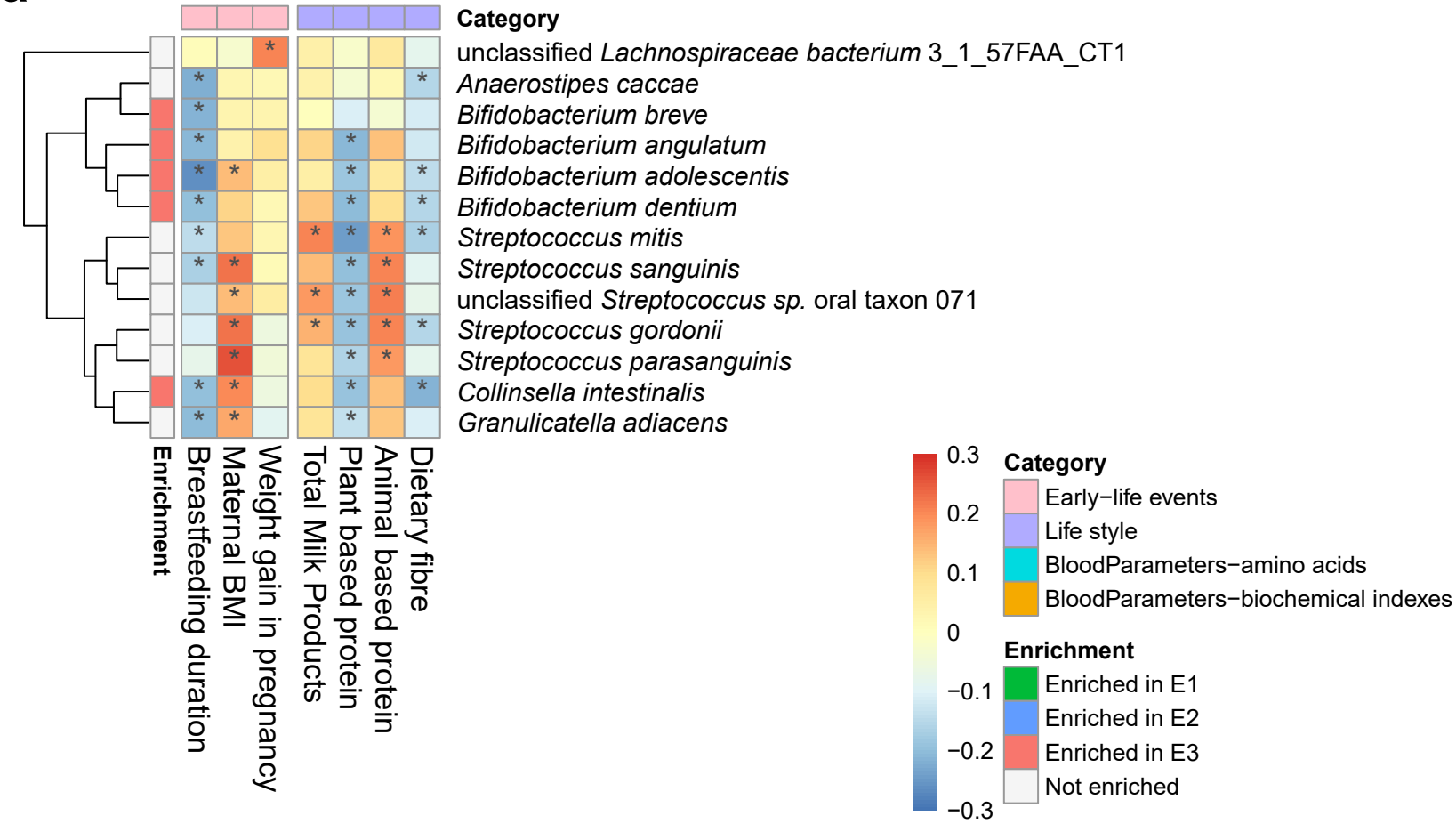

b

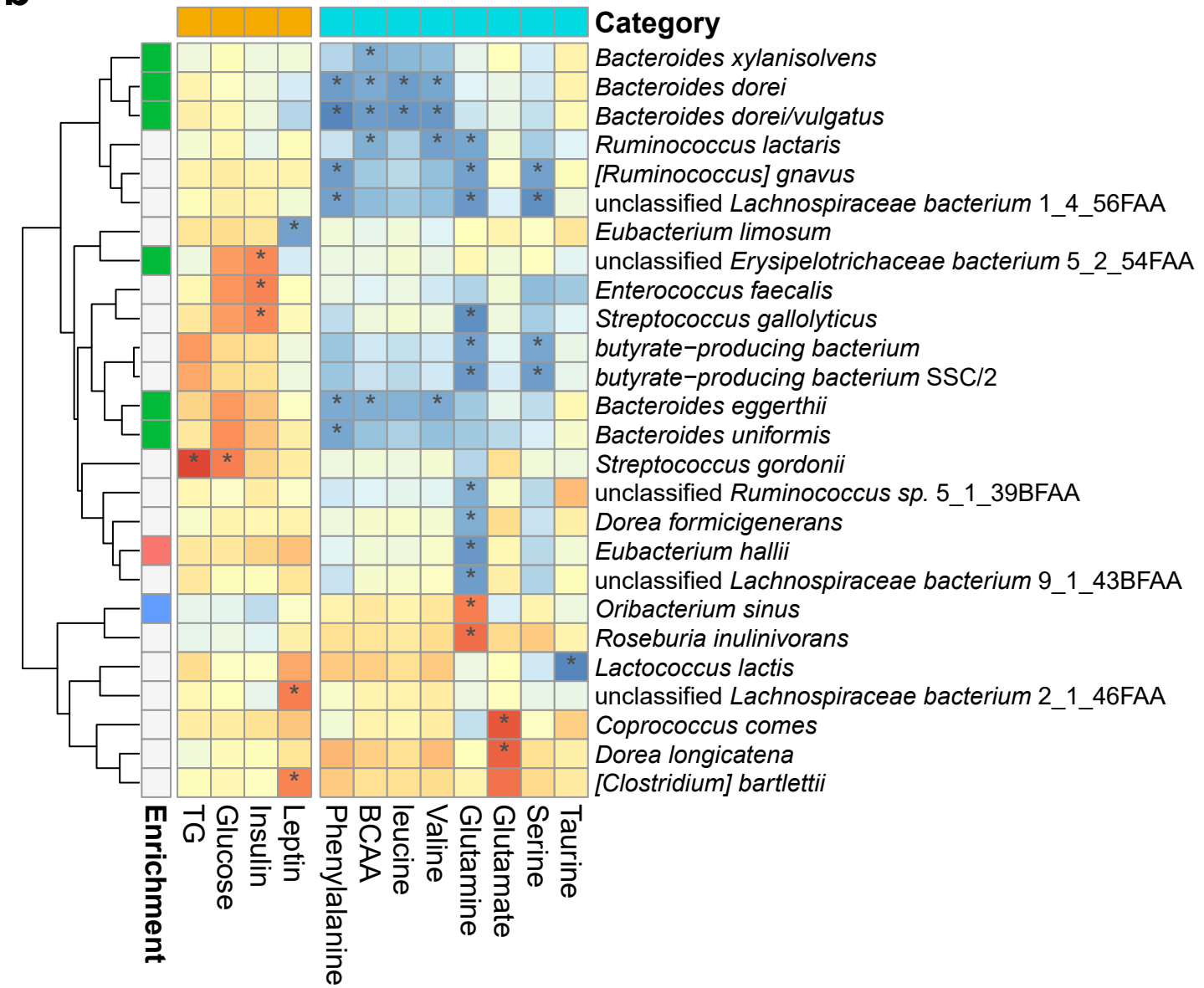

Supplement: Supplementary file 6 — Figure S5. Correlations between continuous phenotypic parameters and species profile in the entire cohort. (a) Spearman’s rank correlations between early events, pre-school lifestyle and species profile (n = 281). (b) Spearman’s rank correlations between blood parameters and species profiles (n = 281). P values were adjusted for each parameter. The “*” indicates significant correlation with adjusted P < 0.05. Species significantly correlated with at least one factor are presented. (PDF 301 kb) [file 40168_2018_608_MOESM6_ESM.pdf]

**a**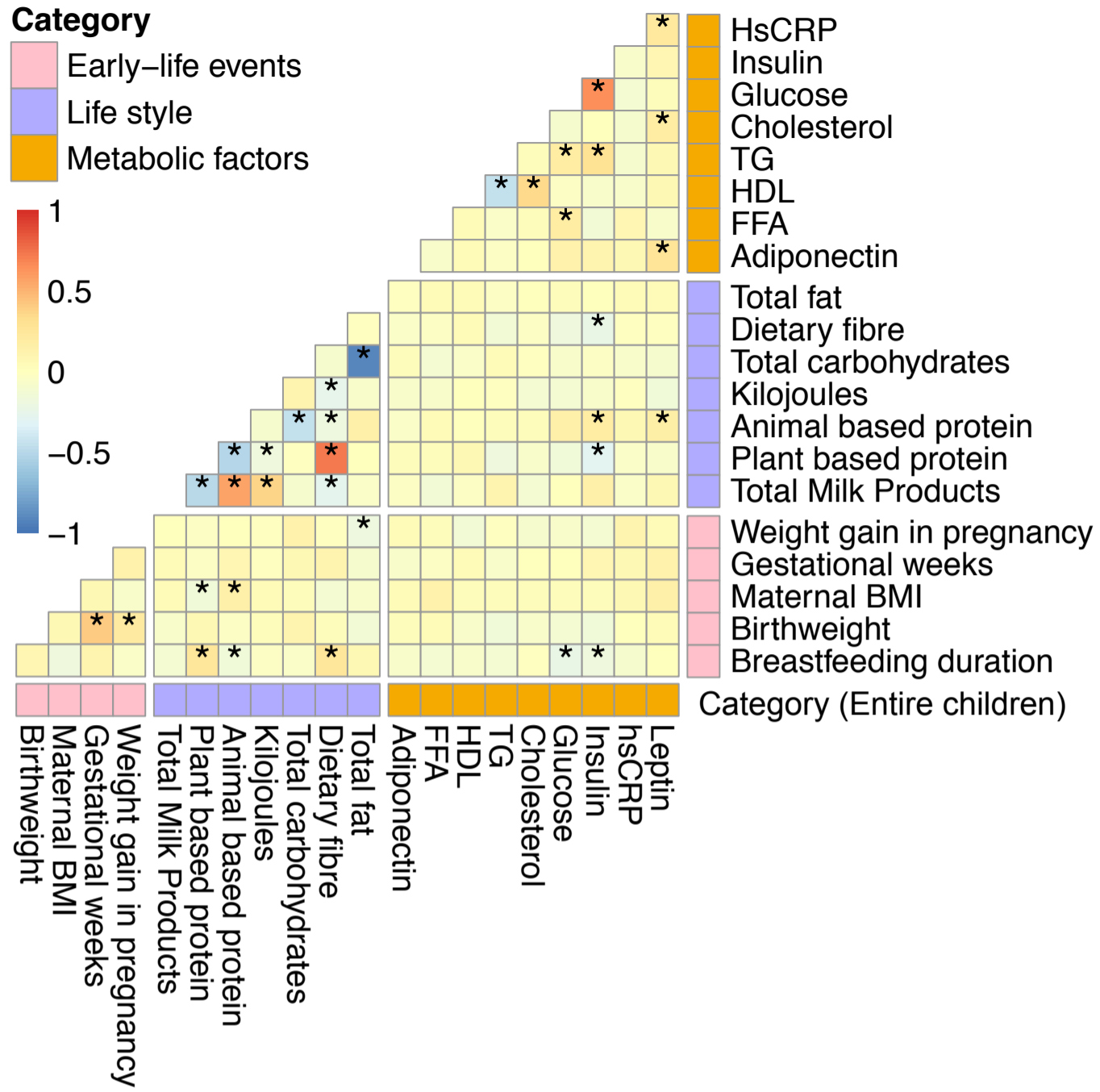**b**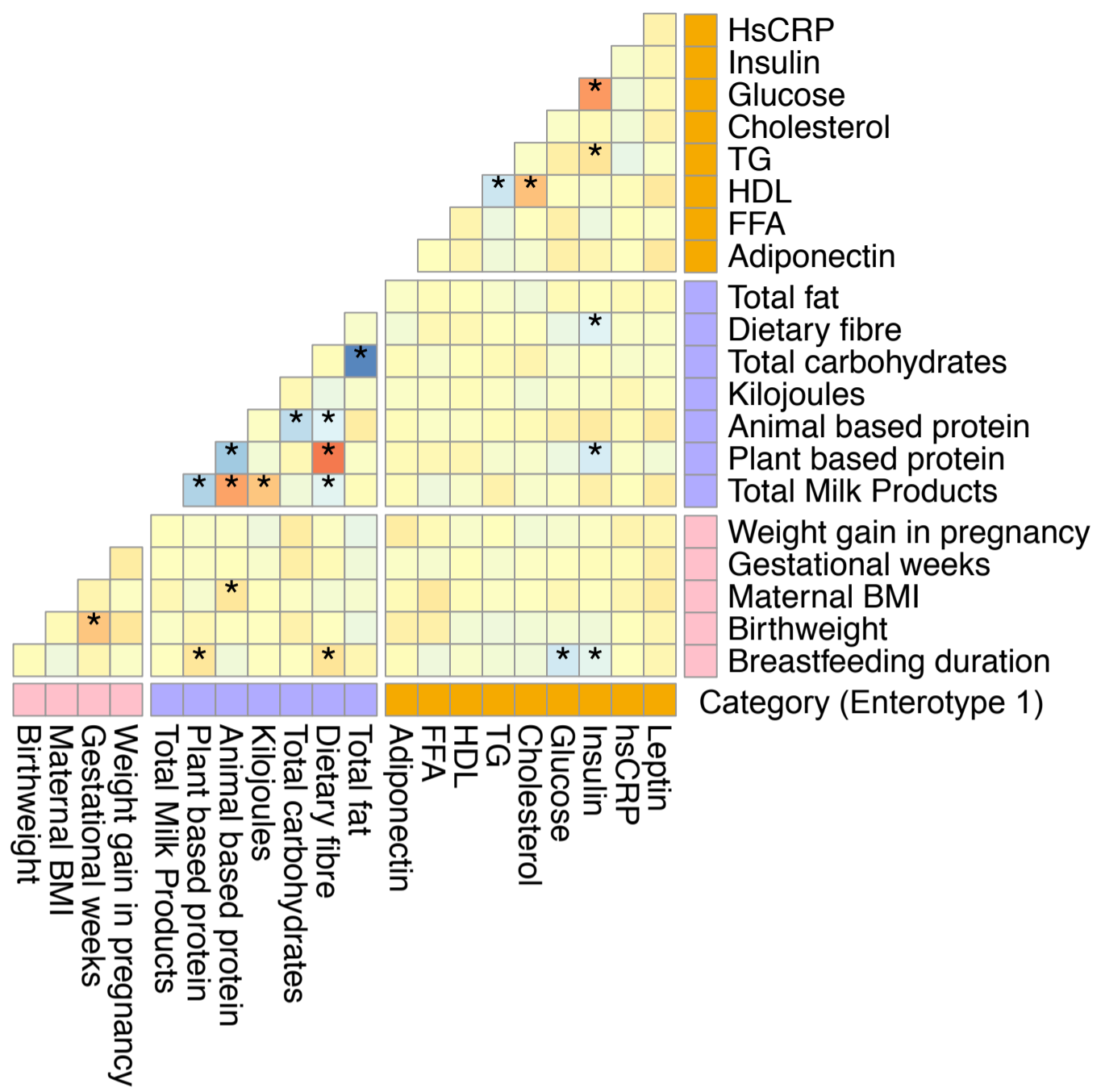**c**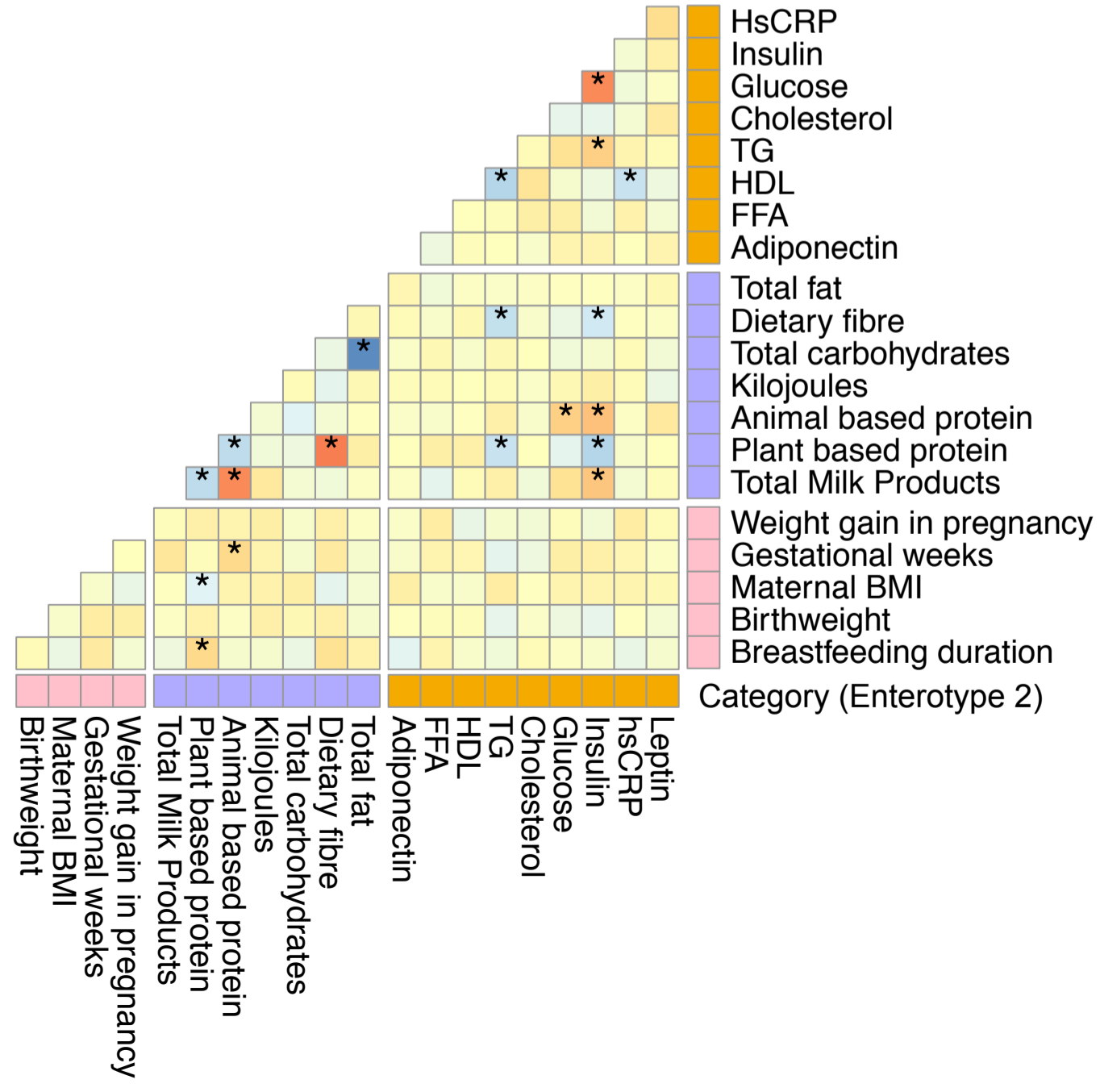**d**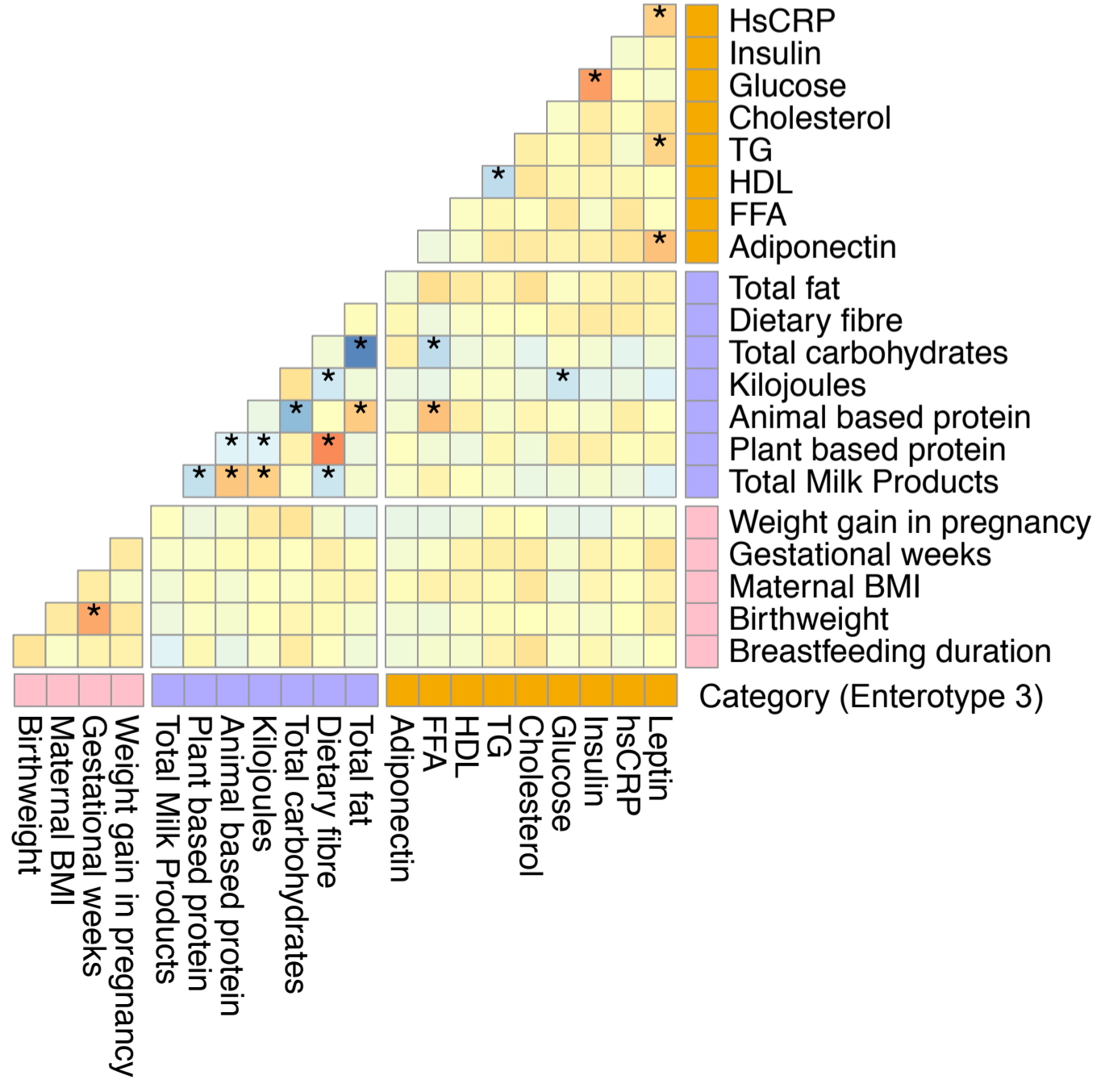

Supplement: Supplementary file 7 — Figure S6. Correlations between continuous phenotypic parameters. (a) Spearman’s rank correlations between continuous phenotypic parameters in the entire cohort (n = 281). (b) Spearman’s correlations between continuous phenotypic parameters in E1 (n = 143). (c) Spearman’s rank correlations between continuous phenotypic parameters in E2 (n = 74). (d) Spearman’s rank correlations between continuous phenotypic parameters in E3 (n = 64). The “*” indicates significant correlation with adjusted P < 0.05 (PDF 296 kb) [file 40168_2018_608_MOESM7_ESM.pdf]

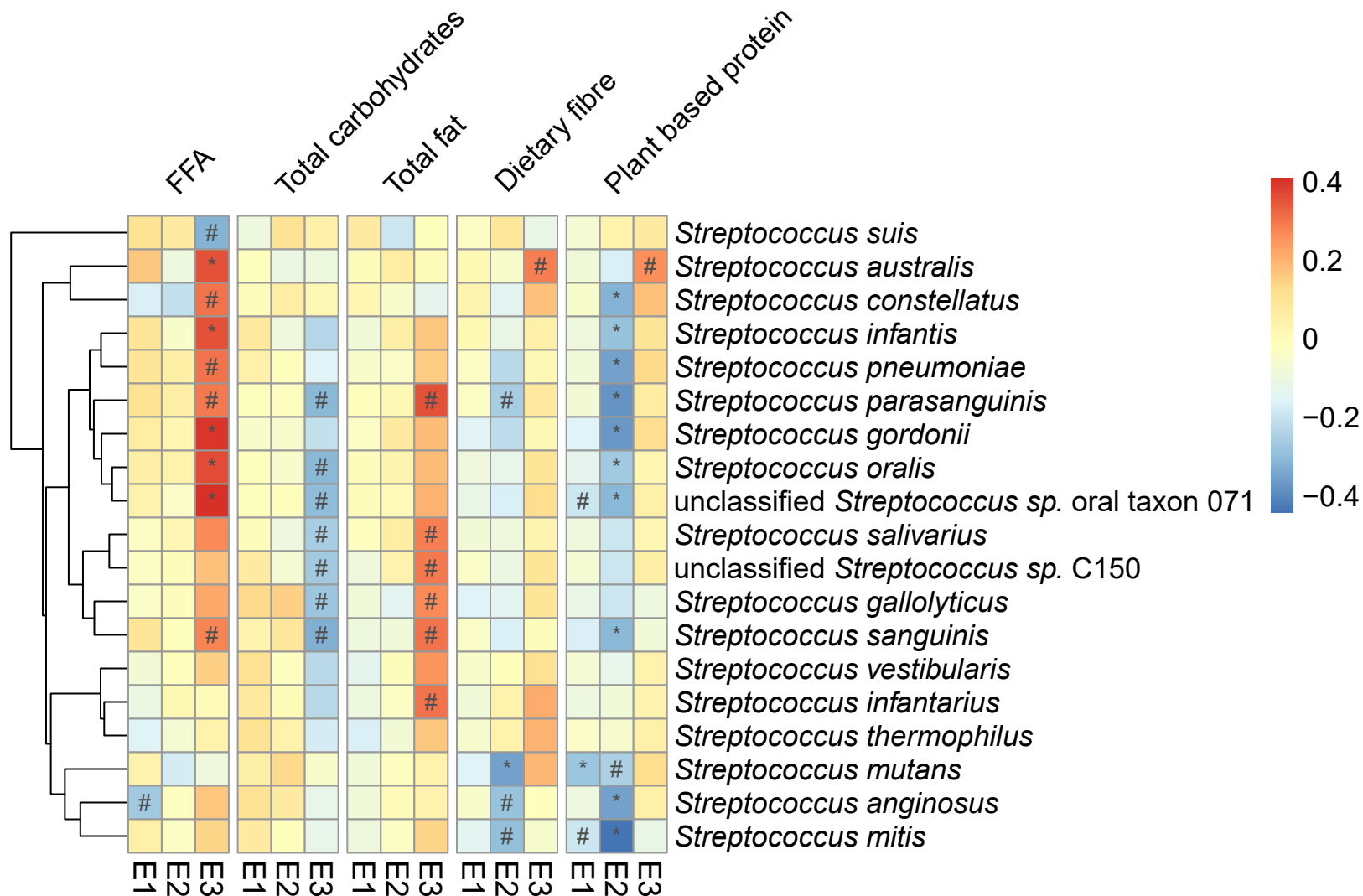

Supplement: Supplementary file 8 — Figure S7. Correlations between Streptococcus species profile and selected phenotypic parameters in enterotypes. Heatmap showing the Spearman’s rank correlations between Streptococcus species and selected phenotypic parameters including free fatty acids levels and the intake of total carbohydrate, total fat, dietary fiber, and plant-based protein. P values were adjusted for each parameter. The “*” indicates significant correlation with adjusted P < 0.05. The “#” indicates correlation with P < 0.05 and adjusted P > 0.05. FFA, free fatty acids. (PDF 197 kb) [file 40168_2018_608_MOESM8_ESM.pdf]

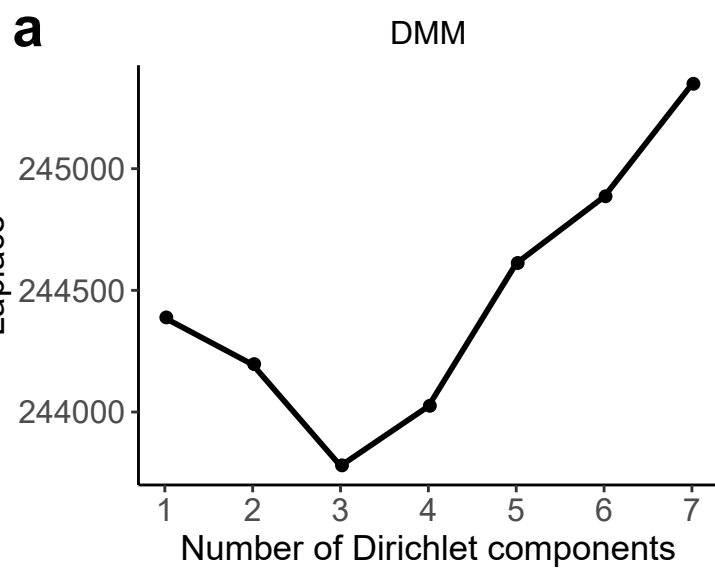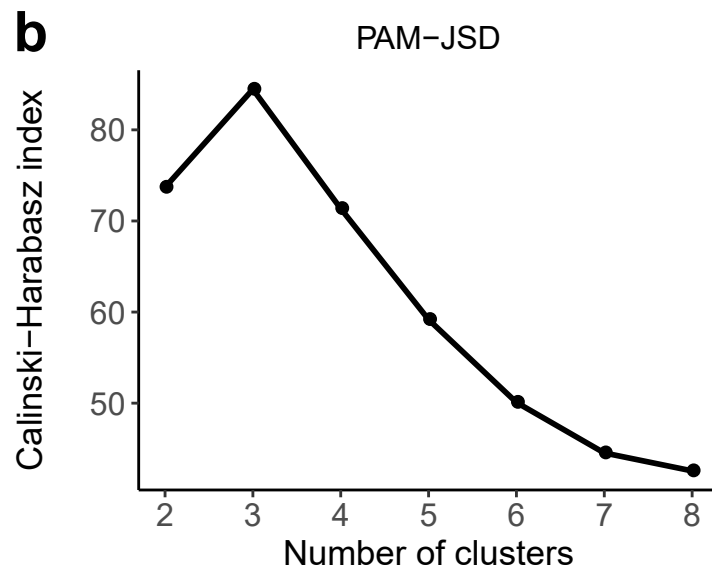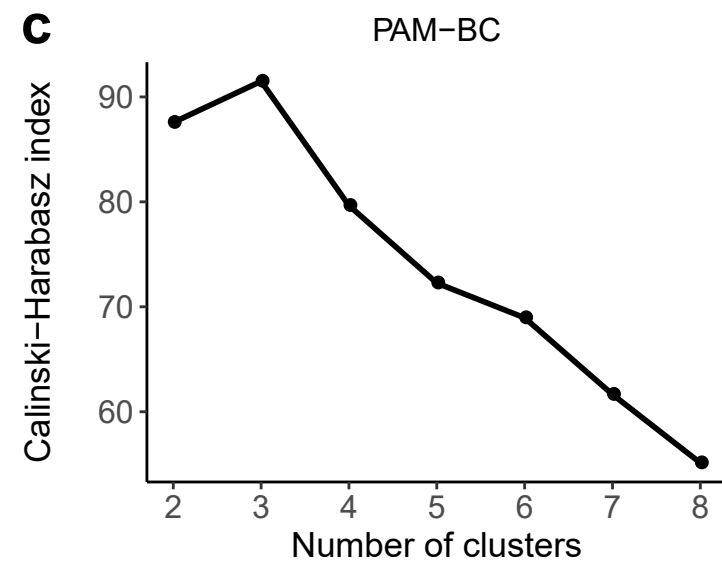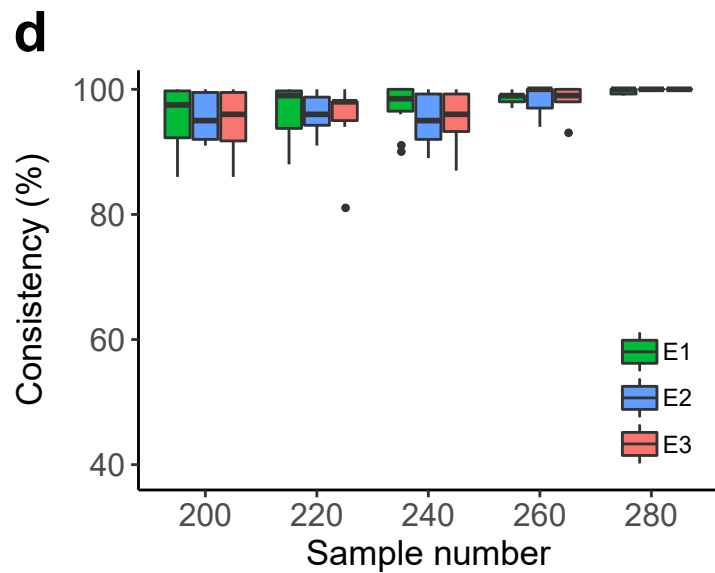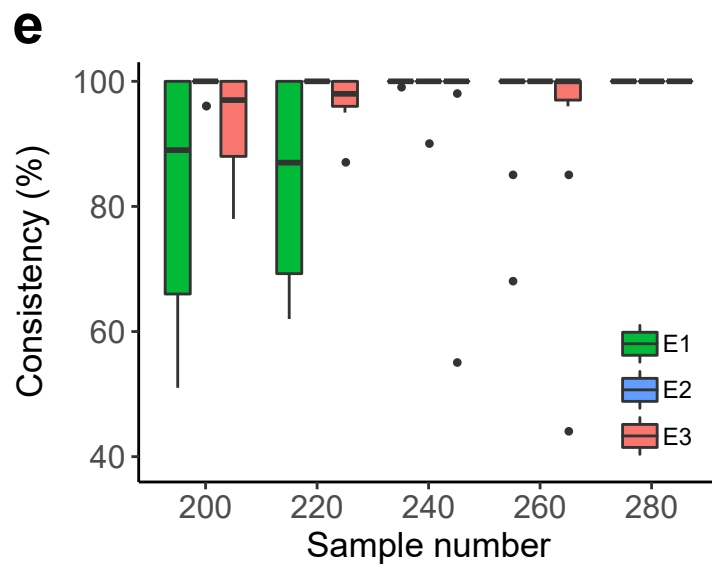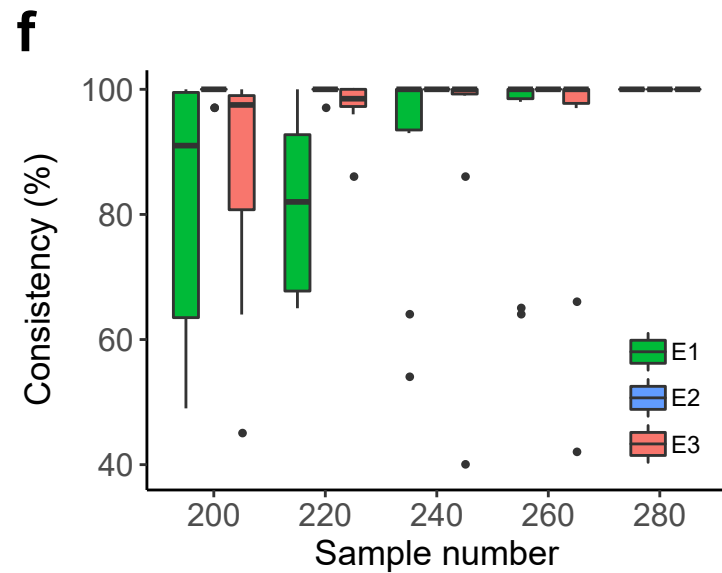

Supplement: Supplementary file 9 — Figure S8. Evaluation of enterotying protocols for Dutch children. (a-c) Evaluation of optimal cluster number by using the DMM protocol (a), the PAM-JSD protocol (b) and the PAM-BC protocol (c). The optimal number of clusters was calculated using Laplace approximation for the DMM protocol (a) and the Calinski–Harabasz index for the PAM-based protocols (b-c). Cluster stability using the DMM (d), the PAM-JSD (e) and the PAM-BC protocols (f). The X axis indicates resampling number and the Y axis indicates the consistency of resampling relative to the original result based on 281 samples. (PDF 221 kb) [file 40168_2018_608_MOESM9_ESM.pdf]
