# Supplementary material for: Impact of early events and lifestyle on the gut microbiota and metabolic phenotypes in young school-age children
Source: Microbiome. 2019 Jan 4;7:2. doi: 10.1186/s40168-018-0608-z (PMC6320620; doi:10.1186/s40168-018-0608-z)
Supplement: Supplementary file 5 — Figure S4. Comparison of gut microbial functional potentials between enterotypes. (a) Differentially enrichment of KEGG modules between enterotypes. Dashed lines indicate a reporter score of 1.96, corresponding to 95% confidence in a normal distribution. (b) Heatmap showing that the relative abundance profiles of 8 selected KOs involved in key functions of metabolic pathways for carbohydrate metabolism (K00845, K01051 and K00873) and amino acid biosynthesis (K01738, K00928, K00058, K00651and K00765) distinguishes E3 from E1 and E2. (PDF 358 kb) [file 40168_2018_608_MOESM5_ESM.pdf]

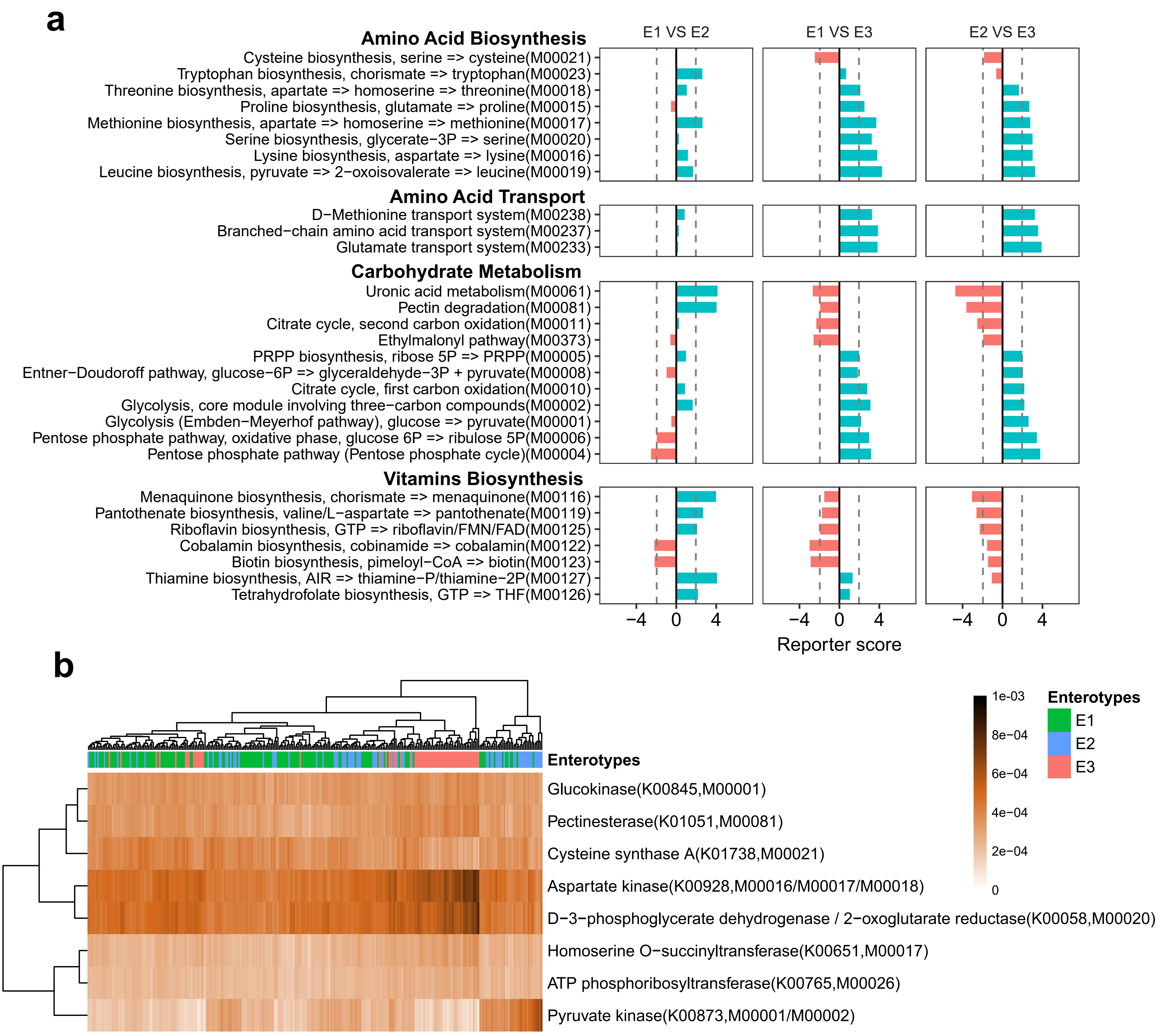

a
Amino Acid Biosynthesis
E1 VS E2
E1 VS E3
E2 VS E3
Cysteine biosynthesis, serine => cysteine(M00021)
Tryptophan biosynthesis, chorismate => tryptophan(M00023)
Threonine biosynthesis, apartate => homoserine => threonine(M00018)
Proline biosynthesis, glutamate => proline(M00015)
Methionine biosynthesis, apartate => homoserine => methionine(M00017)
Serine biosynthesis, glycerate−3P => serine(M00020)
Lysine biosynthesis, aspartate => lysine(M00016)
Leucine biosynthesis, pyruvate => 2−oxoisovalerate => leucine(M00019)
Amino Acid Transport
D−Methionine transport system(M00238)
Branched−chain amino acid transport system(M00237)
Glutamate transport system(M00233)
Carbohydrate Metabolism
Uronic acid metabolism(M00061)
Pectin degradation(M00081)
Citrate cycle, second carbon oxidation(M00011)
Ethylmalonyl pathway(M00373)
PRPP biosynthesis, ribose 5P => PRPP(M00005)
Entner−Doudoroff pathway, glucose−6P => glyceraldehyde−3P + pyruvate(M00008)
Citrate cycle, first carbon oxidation(M00010)
Glycolysis, core module involving three−carbon compounds(M00002)
Glycolysis (Embden−Meyerhof pathway), glucose => pyruvate(M00001)
Pentose phosphate pathway, oxidative phase, glucose 6P => ribulose 5P(M00006)
Pentose phosphate pathway (Pentose phosphate cycle)(M00004)
Vitamins Biosynthesis
Menaquinone biosynthesis, chorismate => menaquinone(M00116)
Pantothenate biosynthesis, valine/L−aspartate => pantothenate(M00119)
Riboflavin biosynthesis, GTP => riboflavin/FMN/FAD(M00125)
Cobalamin biosynthesis, cobinamide => cobalamin(M00122)
Biotin biosynthesis, pimeloyl−CoA => biotin(M00123)
Thiamine biosynthesis, AIR => thiamine−P/thiamine−2P(M00127)
Tetrahydrofolate biosynthesis, GTP => THF(M00126)
−4 0 4
−4 0 4
−4 0 4
Reporter score
b
Enterotypes
Glucokinase(K00845,M00001)
Pectinesterase(K01051,M00081)
Cysteine synthase A(K01738,M00021)
Aspartate kinase(K00928,M00016/M00017/M00018)
D−3−phosphoglycerate dehydrogenase / 2−oxoglutarate reductase(K00058,M00020)
Homoserine O−succinyltransferase(K00651,M00017)
ATP phosphoribosyltransferase(K00765,M00026)
Pyruvate kinase(K00873,M00001/M00002)
1e-03
8e−04
6e−04
4e−04
2e−04
0
Enterotypes
E1
E2
E3
